# Supplementary material for: Insight into partial agonism by observing multiple equilibria for ligand-bound and Gs-mimetic nanobody-bound β1-adrenergic receptor
Source: Nat Commun. 2017 Nov 27;8:1795. doi: 10.1038/s41467-017-02008-y (PMC5702606; doi:10.1038/s41467-017-02008-y)
Supplement: Supplementary file 1 — Supplementary information [file 41467_2017_2008_MOESM1_ESM.pdf]

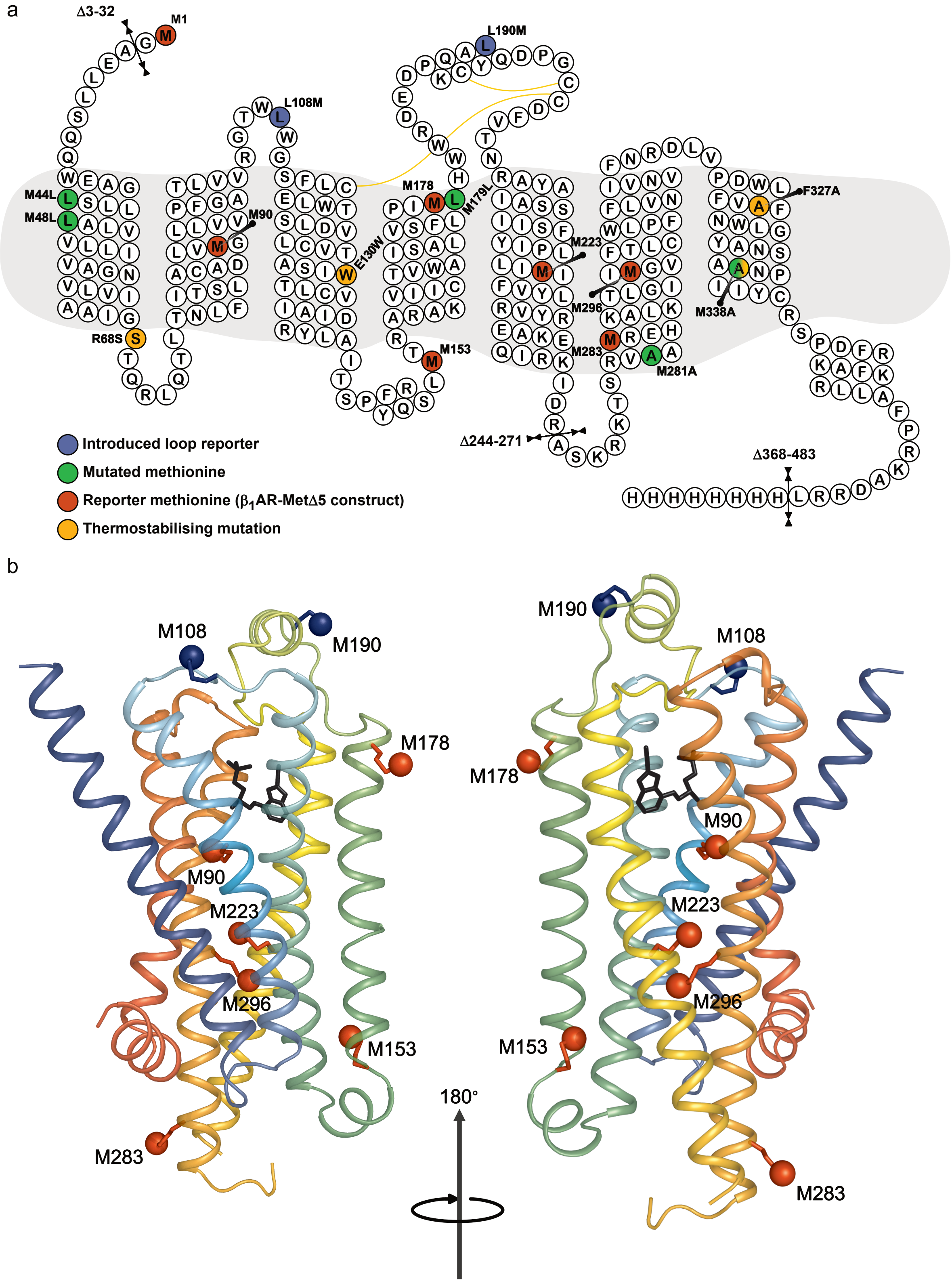
a
Δ3-32
M1
L190M
L108M
M44L
M48L
M90
M178
M179L
E130W
M223
M296
M283
M281A
M153
R68S
F327A
M338A
Δ244-271
Δ368-483
Introduced loop reporter
Mutated methionine
Reporter methionine (β1AR-MetΔ5 construct)
Thermostabilising mutation
b
M108
M190
M178
M90
M223
M296
M153
M283
180°

Supplementary Figure 1: Location of the methionine residues in the turkey β_1AR construct used in the NMR studies.

(a) Secondary structure diagram for the turkey β_1AR-MetΔ5 receptor used in this study. The construct was derived from the truncated β_1AR receptor β44-m23[14] through reversal of three stabilising mutations to WT (see Methods) and the mutation of five methionine residues to reduce the spectral overlap. The remaining native methionine residues are shown in red with the three thermostabilising mutations in yellow. For some of the studies additional methionine reporter residues were introduced into EL2 and EL3 (blue) resulting in the constructs β_1AR-MetΔ5-L108M and β_1AR-MetΔ5-L190M. Deleted stretches of the receptor sequence at the N- and C-termini and in IL3 are indicated by arrows and the range of the deletion. Disulphide bonds are shown between connected cysteines in yellow.

(b) Structure of β_1AR bound to cyanopindolol (PDB code 4BVN) shows the methionine residues as red sticks with their methyl groups represented by spheres (red). The receptor backbone trace is shown in ribbon with the cyanopindolol in black stick representation.

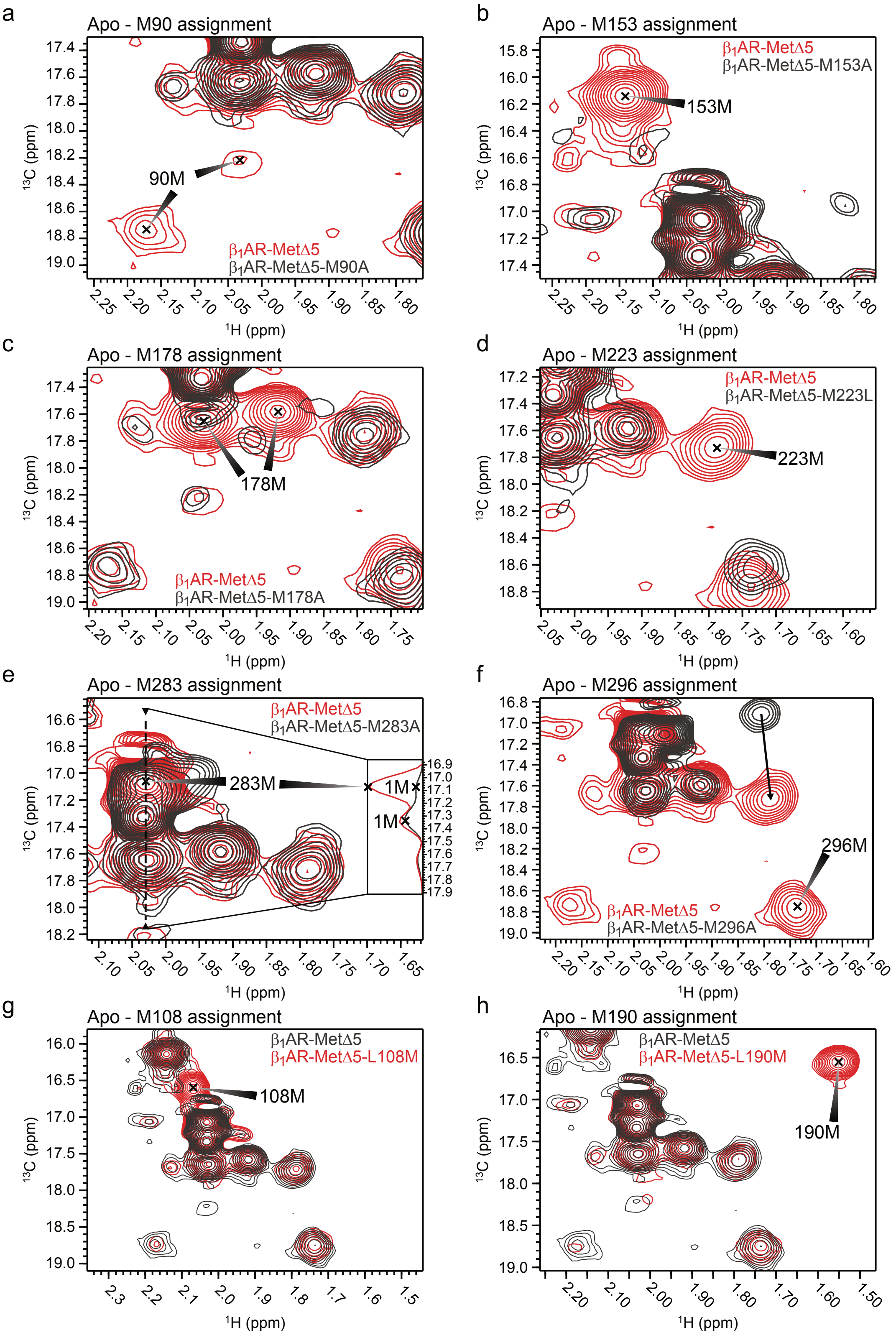
a
Apo - M90 assignment
90M
β1AR-MetΔ5
β1AR-MetΔ5-M90A
b
Apo - M153 assignment
β1AR-MetΔ5
β1AR-MetΔ5-M153A
153M
c
Apo - M178 assignment
178M
β1AR-MetΔ5
β1AR-MetΔ5-M178A
d
Apo - M223 assignment
β1AR-MetΔ5
β1AR-MetΔ5-M223L
223M
e
Apo - M283 assignment
β1AR-MetΔ5
β1AR-MetΔ5-M283A
283M
1M
1M
f
Apo - M296 assignment
296M
β1AR-MetΔ5
β1AR-MetΔ5-M296A
g
Apo - M108 assignment
β1AR-MetΔ5
β1AR-MetΔ5-L108M
108M
h
Apo - M190 assignment
β1AR-MetΔ5
β1AR-MetΔ5-L190M
190M
13C (ppm)
1H (ppm)

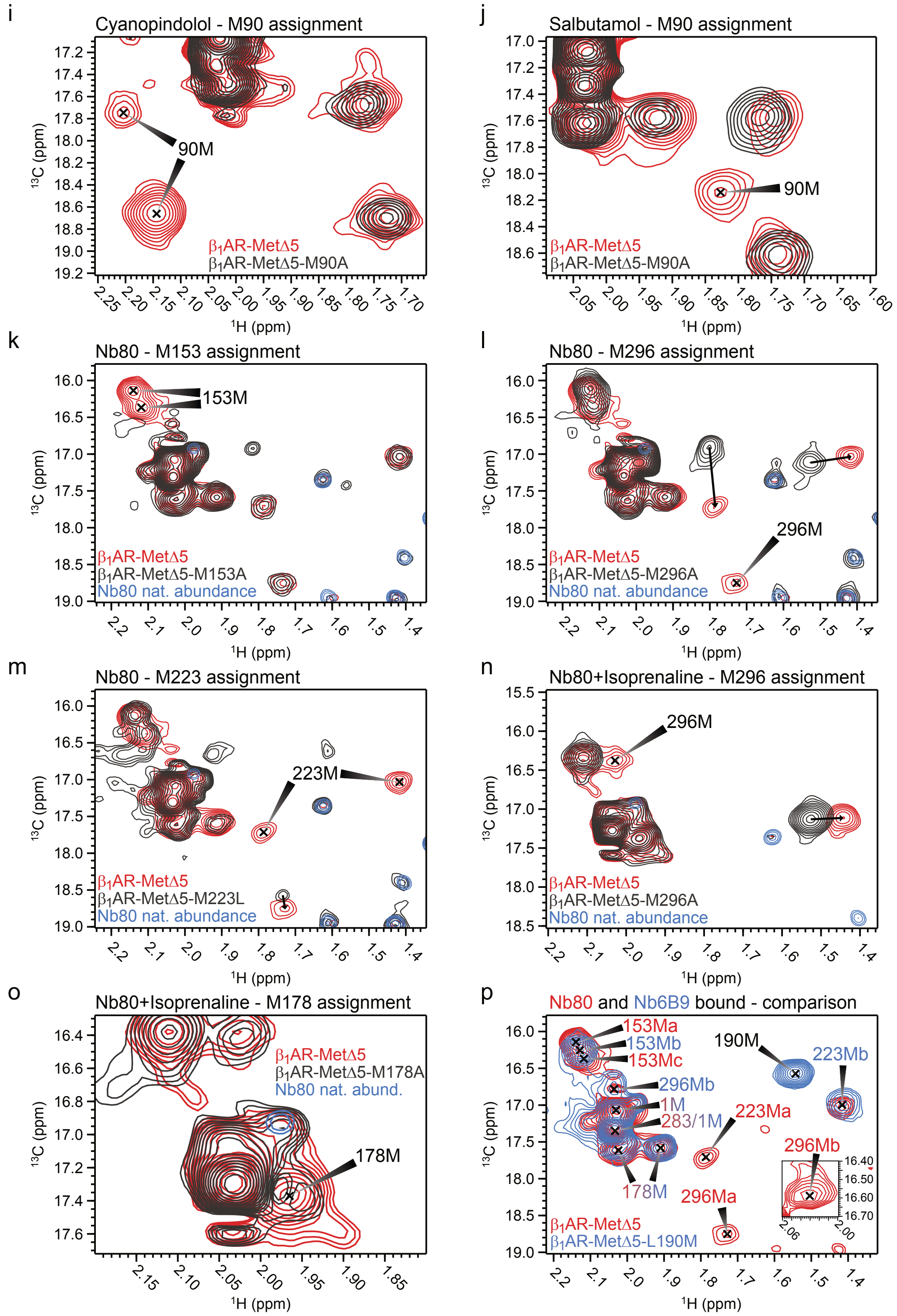

i
Cyanopindolol - M90 assignment
90M
β1AR-MetΔ5
β1AR-MetΔ5-M90A
13C (ppm)
1H (ppm)
j
Salbutamol - M90 assignment
90M
β1AR-MetΔ5
β1AR-MetΔ5-M90A
k
Nb80 - M153 assignment
153M
β1AR-MetΔ5
β1AR-MetΔ5-M153A
Nb80 nat. abundance
l
Nb80 - M296 assignment
296M
β1AR-MetΔ5
β1AR-MetΔ5-M296A
Nb80 nat. abundance
m
Nb80 - M223 assignment
223M
β1AR-MetΔ5
β1AR-MetΔ5-M223L
Nb80 nat. abundance
n
Nb80+Isoprenaline - M296 assignment
296M
β1AR-MetΔ5
β1AR-MetΔ5-M296A
Nb80 nat. abundance
o
Nb80+Isoprenaline - M178 assignment
178M
β1AR-MetΔ5
β1AR-MetΔ5-M178A
Nb80 nat. abund.
p
Nb80 and Nb6B9 bound - comparison
153Ma
153Mb
153Mc
190M
223Mb
296Mb
1M
283/1M
223Ma
296Mb
178M
296Ma
β1AR-MetΔ5
β1AR-MetΔ5-L190M

Supplementary Figure 2: Assignment of ^{13}C methyl labelled methionine residues in ^{1}H,^{13}C SOFAST HMQC spectra by mutagenesis.
Identical experiments were recorded with respect to the presence of ligands or nanobody, on β_1AR-MetΔ5 and a range of β_1AR-MetΔ6 constructs, where an additional methionine was mutated. The resulting absence of a peak or peaks in the β_1AR-MetΔ6 comparison spectrum allowed the assignment of those peaks in the β_1AR-MetΔ5 spectrum to the mutated methionine. (a) to (o) shows all the unique assignment spectra of individual residues in the apo (a–h) form, cyanopindolol- (i), salbutamol- (j), Nb80- (k–m) and Nb80- and isoprenaline-bound forms (n,o). β_1AR-MetΔ5 is shown in red and β_1AR-MetΔ6 is shown in black, highlighting the mutated residue. Natural abundance peaks resulting from the presence of Nb80 are shown in blue (k–o). It was possible to unambiguously transfer the residue assignment here obtained to receptor states not shown above. The signal from the N-terminal methionine (1M) overlaps with Met283$^{6.28}$, however a cross section at 2.03 ppm in the ^{1}H dimension ((e), inset), reveals a clear contribution from Met283$^{6.28}$. The spatial proximity of methionines 223$^{5.54}$ and 296$^{6.41}$ means that the mutation of either one causes chemical shift changes in the signal from the other. Such changes are highlighted with black arrows (f, l–n).

As all nanobody-bound assignment spectra were recorded with Nb80, the transferability of assignments to Nb6B9-bound receptor is shown in (p). Lower affinity Nb80-bound β_1ARΔ5 is shown in red and high affinity Nb6B9-bound β_1ARΔ5-L190M is shown in blue. Methionines 153 and 223$^{5.54}$ show two peaks in the Nb80-bound spectrum, as the amount of Nb80 present is non-saturating, in contrast to Nb6B9. For the latter, the whole population of Met223$^{5.54}$ transitions to chemical shift position 223Mb in the presence of Nb6B9, from the apo form population at chemical shift 223Ma. Similarly Met153 shows as a single peak in the presence of Nb6B9 at position 153Mb, having transitioned from the apo state position 153Ma. The scale of this chemical shift transition is greater in the presence of Nb80 shown as position 153Mc. For Met296$^{6.41}$ a similar trend is observed, however the non-apo end-state at position 296Mb is only observed with Nb6B9, where the nanobody is present in saturating amounts. In the presence of saturating amounts of Nb80, Met296$^{6.41}$ shows as a single peak near position 296Mb ((p) inset 296Mb) with the whole population of 296Ma having completely shifted.

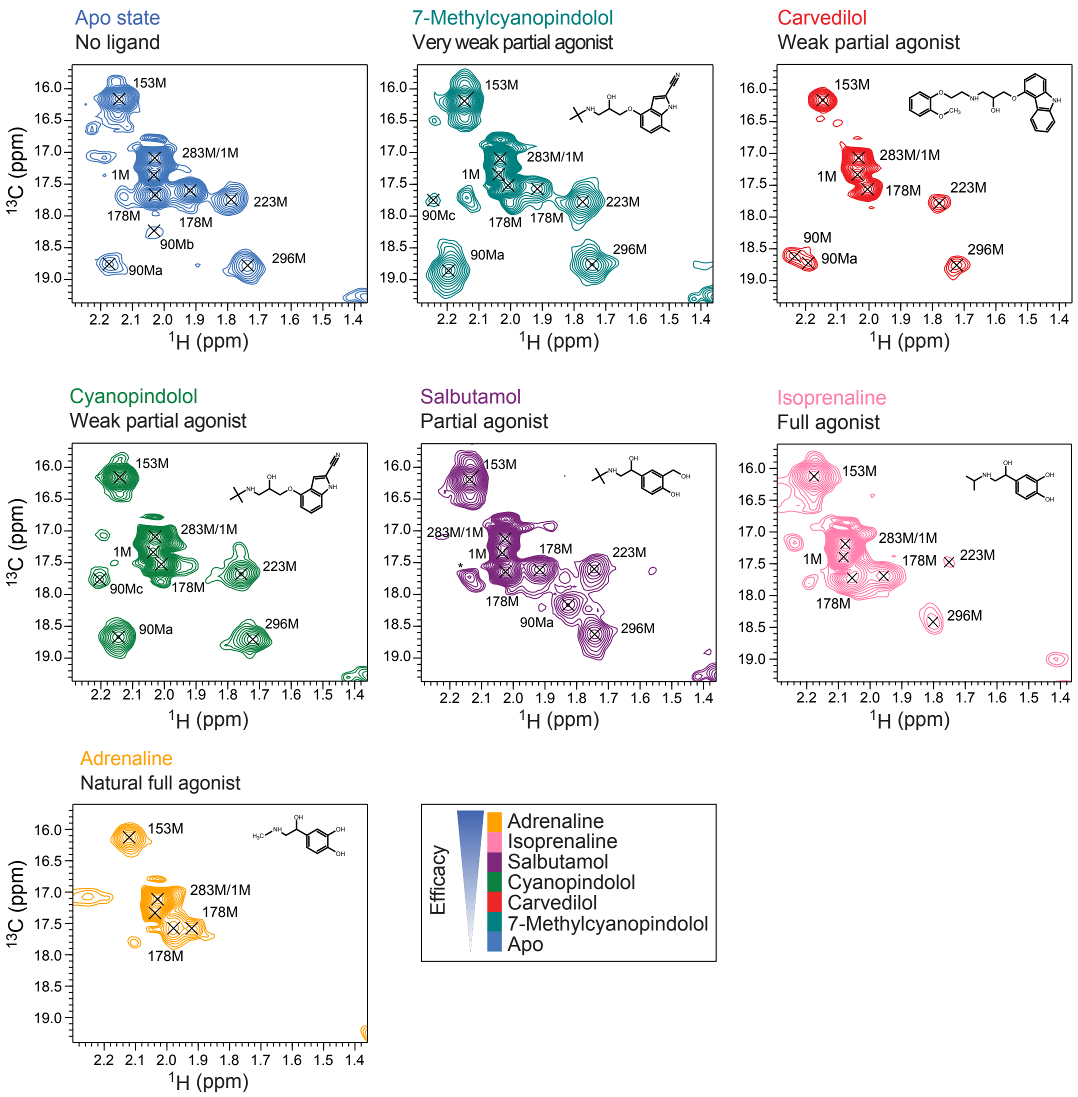

Supplementary Figure 3: Assignment of ligand-bound β_1AR-MetΔ5 methyl methionine resonances.

Methyl region of 2D ^{1}H,^{13}C HMQC spectra with methionine residue assignments for [^{13}C-methyl-Met] β_1AR-MetΔ5 in the apo state and bound to the orthosteric ligands 7-methylcyanopindolol (very weak partial agonist, teal), carvedilol (weak partial agonist, red), cyanopindolol (weak partial agonist, green), salbutamol (partial agonist, purple), isoprenaline (full agonist, pink). Data were recorded at 308 K and 800 MHz (^{1}H) using a gradient coherence order selected ^{1}H,^{13}C SOFAST HMQC experiment (see Methods).

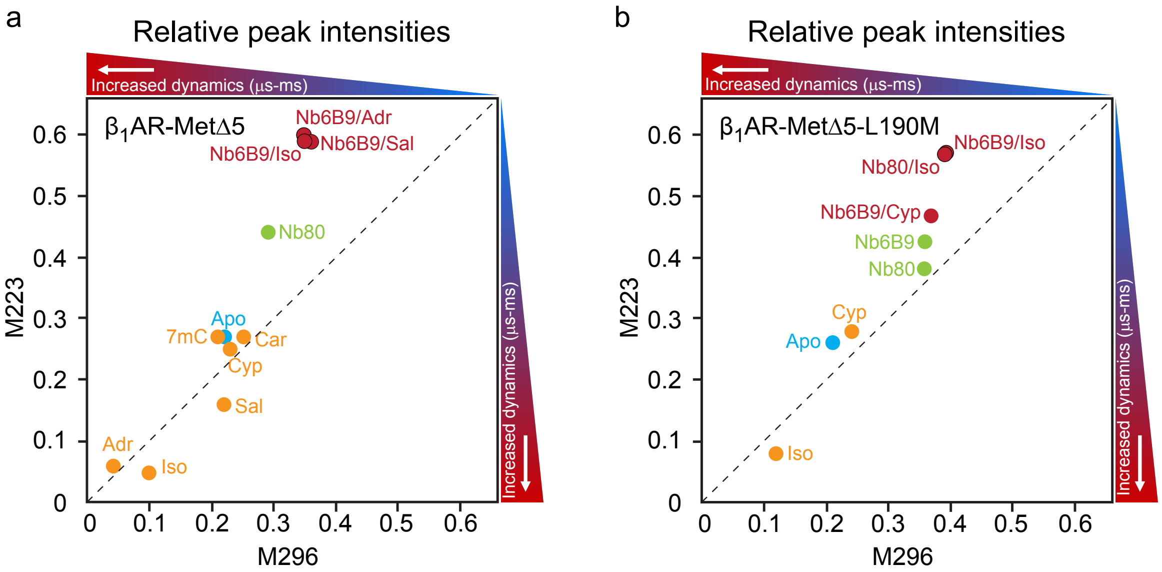

Supplementary Figure 4: Relative signal intensities of M223$^{5.54}$ and M296$^{6.41}$ resonances reflect the dynamic state of the receptor.
The intensities of M223$^{5.54}$ and M296$^{6.41}$ are plotted against each other for the β_1AR-MetΔ5 (a) and β_1AR-MetΔ5-L190M (b) constructs, with the receptor in the apo form (blue), ligand-bound (orange), in the apo form bound to nanobody (green) or ligand-bound in ternary complex with nanobody (red). Relative intensities are measured against the M153 signal as reference. For M223$^{5.54}$ and M296$^{6.41}$ a similar intensity pattern is observed as for the normalised peak intensities shown in Fig. 2. The ternary (red) and nanobody-bound ligand-free (green) complexes show the highest relative intensities corresponding to a less dynamic receptor state while the ligand-bound receptors and the apo form are more dynamic, resulting in lower intensities due to dynamics that lead to broader signals. The intensities of full agonist-bound β_1AR receptor with isoprenaline or adrenaline are particularly low, indicating extensive conformational dynamics that result in substantial signal broadening. For nanobody-bound receptor (basal and ternary complexes) M296$^{6.41}$ remains more dynamic on the slow μs-to-ms timescale than M223$^{5.54}$ indicating that there is residual motion in TM6 even in the fully active receptor form. The coloured bars on the top and the right hand side of each graph indicate the direction of increasing dynamics, with red indicative of highly dynamic states (μs-ms timescale) and blue representative of less dynamic, more rigid receptor states.

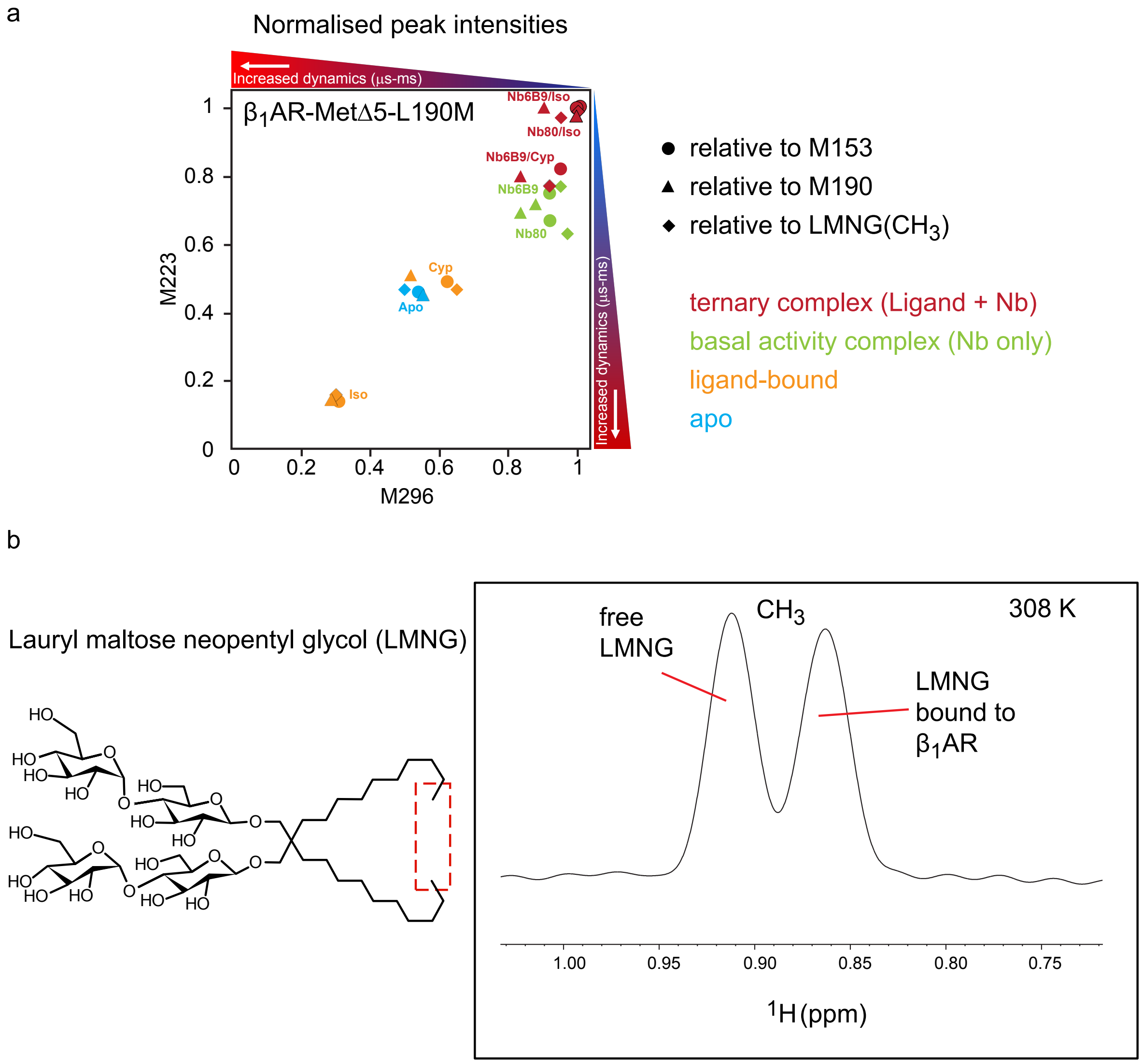

Supplementary Figure 5: Intensity data for $M223^{5.54}$ and $M296^{6.41}$ resonances provide a robust proxy for the assessment of receptor dynamics. (a) A comparison of the intensity analysis for $M223^{5.54}$ and $M296^{6.41}$ using different signals as intensity reference: The analysis was performed relative to M153, M190 and the protein-bound methyl group signal of LMNG (see b). Based on their location on the structure (M153 IL2, M190 EL2, LMNG) and their properties in the spectra it was assumed that all three residues are relatively free of slower dynamics and hence should be suitable as a reference for a methyl group free of slow conformational dynamics. Indeed, for all three reference signals the same overall trends in intensity are observed transitioning from full agonist-bound receptor (isoprenaline and adrenaline) at low relative intensities through the apo form and other orthosteric ligand-bound forms at intermediate relative intensities, through to higher intensities for ternary complexes and

nanobody-bound β_1AR. The coloured bars on the top and the right hand side of the graph indicate the direction of increasing dynamics, with red indicative of highly dynamic states (µs-ms timescale) and blue representative of less dynamic, more rigid receptor states.

(b) LMNG reference signal: A ^{1}H NMR spectrum of the alkyl side chain methyl group signal of LMNG (structure shown) shows the detergent is in slow exchange between receptor-bound form and free form. Depending on the temperature, the amount of protein-bound detergent varies between 40 to 45 molecules per receptor. The signal can be used for chemical shift referencing, as an intensity reference and to assess the sample quality.

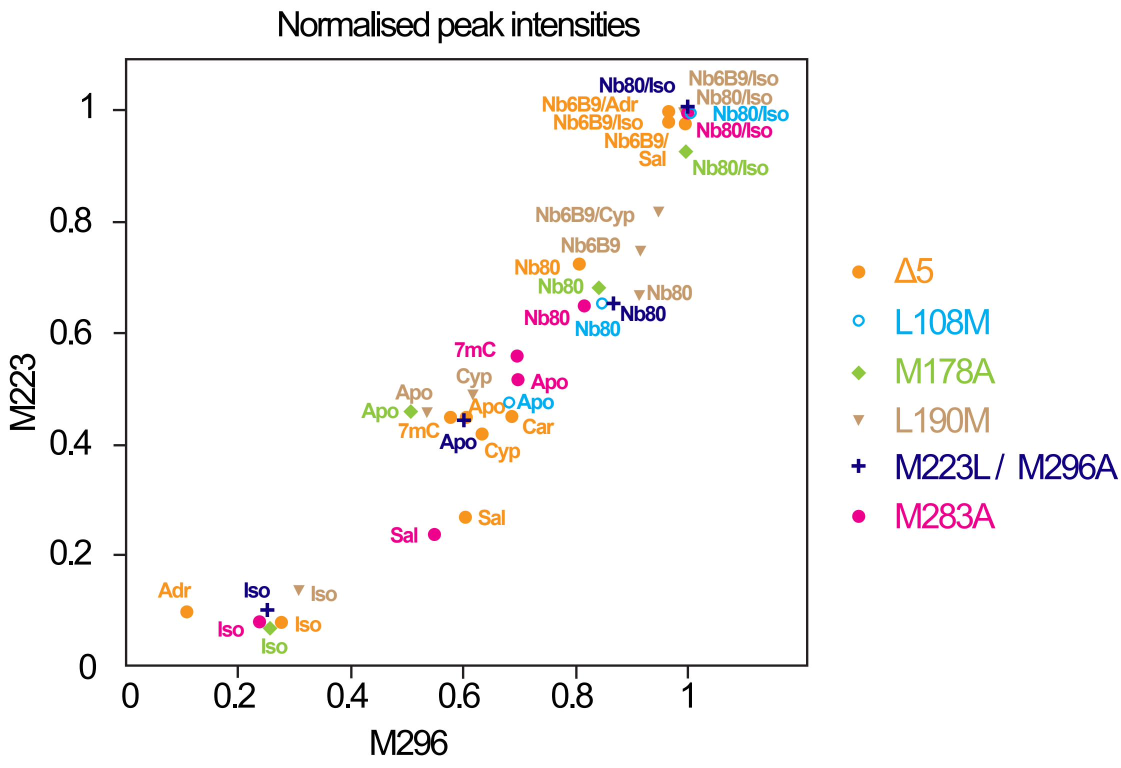

Supplementary Figure 6: Intensity data of M223$^{5.54}$ and M296$^{6.41}$ resonances from different β_1AR constructs reveal a consistent picture.

Normalised intensities for M223$^{5.54}$ and M296$^{6.41}$ are shown for various constructs used in this work: β_1AR-MetΔ5 (orange), β_1AR-MetΔ5-L108M (cyan), β_1AR-MetΔ5-L178M (green), β_1AR-MetΔ5-L190M (tan), β_1AR-MetΔ5-M283A (pink) and β_1AR-MetΔ5-M223L / β_1AR-MetΔ5-M296A (navy). The last two constructs are displayed as a combined set. Again, a consistent pattern is seen with ternary and nanobody-bound complexes found at the highest normalised intensities, ligand-bound and apo forms at intermediate values and full agonist-bound forms at the lowest intensities, corroborating the trends of increasing dynamics shown earlier for β_1AR-MetΔ5 and β_1AR-MetΔ5-L190M.

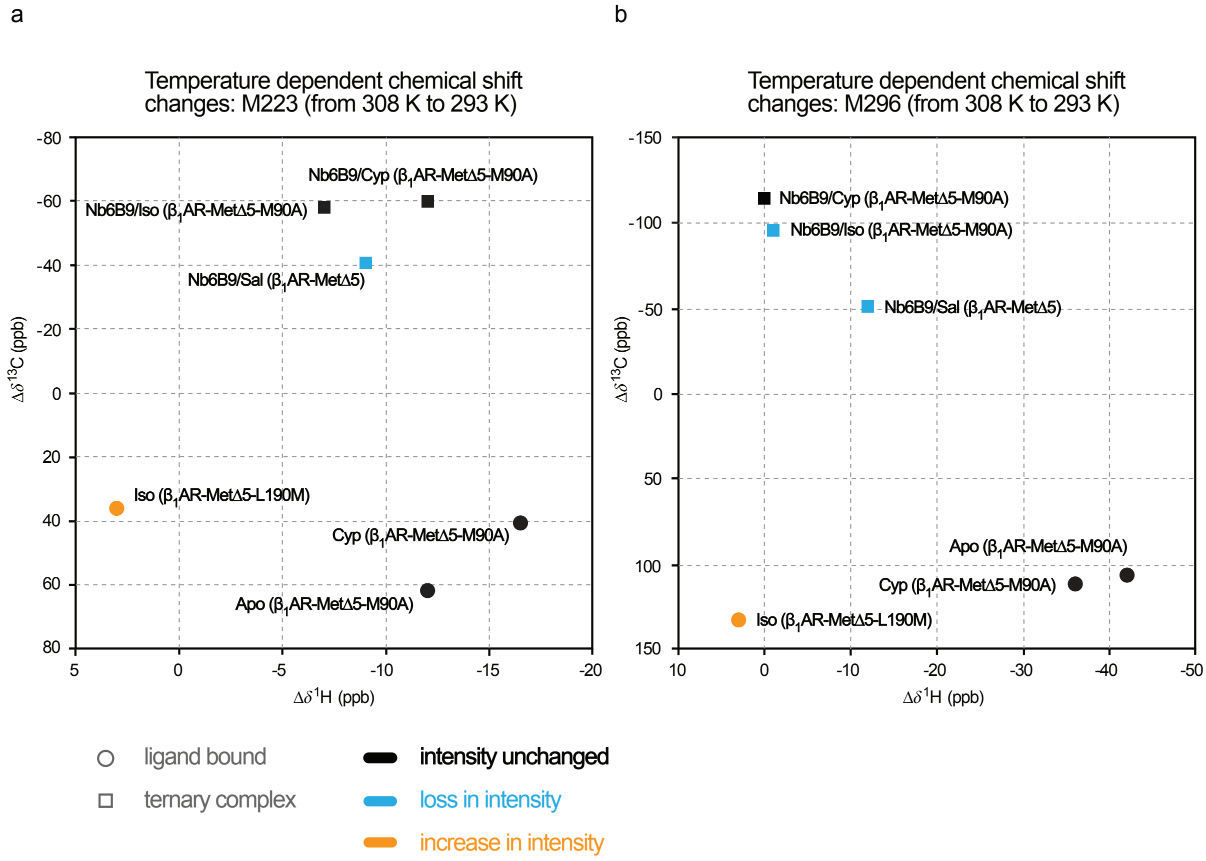

Supplementary Figure 7: Temperature-dependent chemical shift changes for M223$^{5.54}$ and M296$^{6.41}$ resonances in ligand-bound and ternary complex spectra.

Graphical representation of ^{1}H,^{13}C HMQC data of chemical shift changes for M223$^{5.54}$ (a) and M296$^{6.41}$ (b) resonances as the temperature is lowered from 308 K to 293 K. The change in ^{1}H chemical shift ($\Delta\delta$ ^{1}H) is shown in ppb against the change in ^{13}C chemical shift ($\Delta\delta$ ^{13}C) also in ppb, calculated relative to the peak position at 308 K for each condition i.e. for each condition, the peak position at 308 K corresponds to ($\Delta\delta$ ^{1}H $= 0$, $\Delta\delta$ ^{13}C $= 0$). For example, negative values correspond to a displacement of the resonance to higher field (smaller shifts) at the lower temperature. Peaks in blue indicate a loss in intensity on decreasing the temperature, yellow an increase in intensity and black no change. Circles represent ligand-bound receptor and squares, ternary complex, with the exact ligand and nanobody compositions indicated in the figure. It can be seen that on lowering the temperature ligand-bound complexes move to higher ^{13}C shifts for both M223$^{5.54}$ and M296$^{6.41}$, i.e. towards the trans orientation for χ^3, whilst ternary complexes shift towards lower ^{13}C chemical shifts i.e. towards the gauche orientation of χ^3 (see Supplementary Note 3). Residues showing a loss in intensity

(blue) indicate a shift from fast exchange towards intermediate exchange on the NMR timescale leading to increased peak broadening. Residues showing an increase in intensity likely shift from the intermediate exchange regime (peaks broadened) to slow exchange i.e. where both conformations in the equilibrium can be detected at different chemical shifts with intensities weighted by population. Unchanged intensities suggest no substantial change in the exchange regime relative to the chemical shift difference.

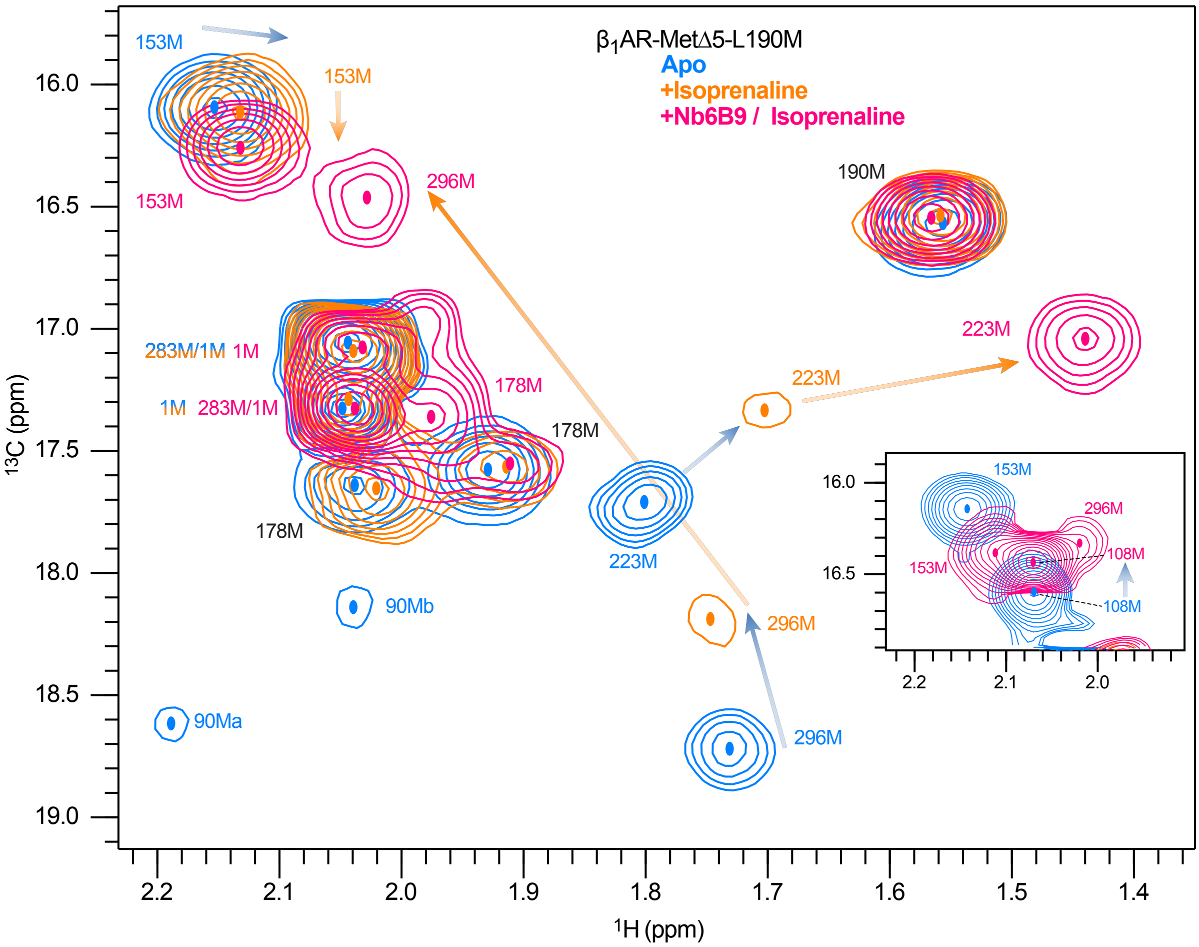

Supplementary Figure 8: Ternary complex formation with isoprenaline and nanobody reveals extensive changes in receptor conformation.

Methyl region of 2D ^{1}H,^{13}C HMQC spectra for [^{13}C-methyl-Met] β_1AR-MetΔ5-L190M showing the chemical shift changes between apo (blue), isoprenaline-bound (orange), and ternary complex receptor bound to Nb6B9 and isoprenaline (pink). The largest chemical shift changes are indicated by arrows. Changes upon formation of the ternary complex are more substantial than those resulting from isoprenaline binding to the apo receptor. Ternary complex formation leads to global changes affecting cytoplasmic as well as extracellular regions of the receptor. The inset shows changes to the M108 resonance in EL1 of β_1AR-MetΔ5-L108M.

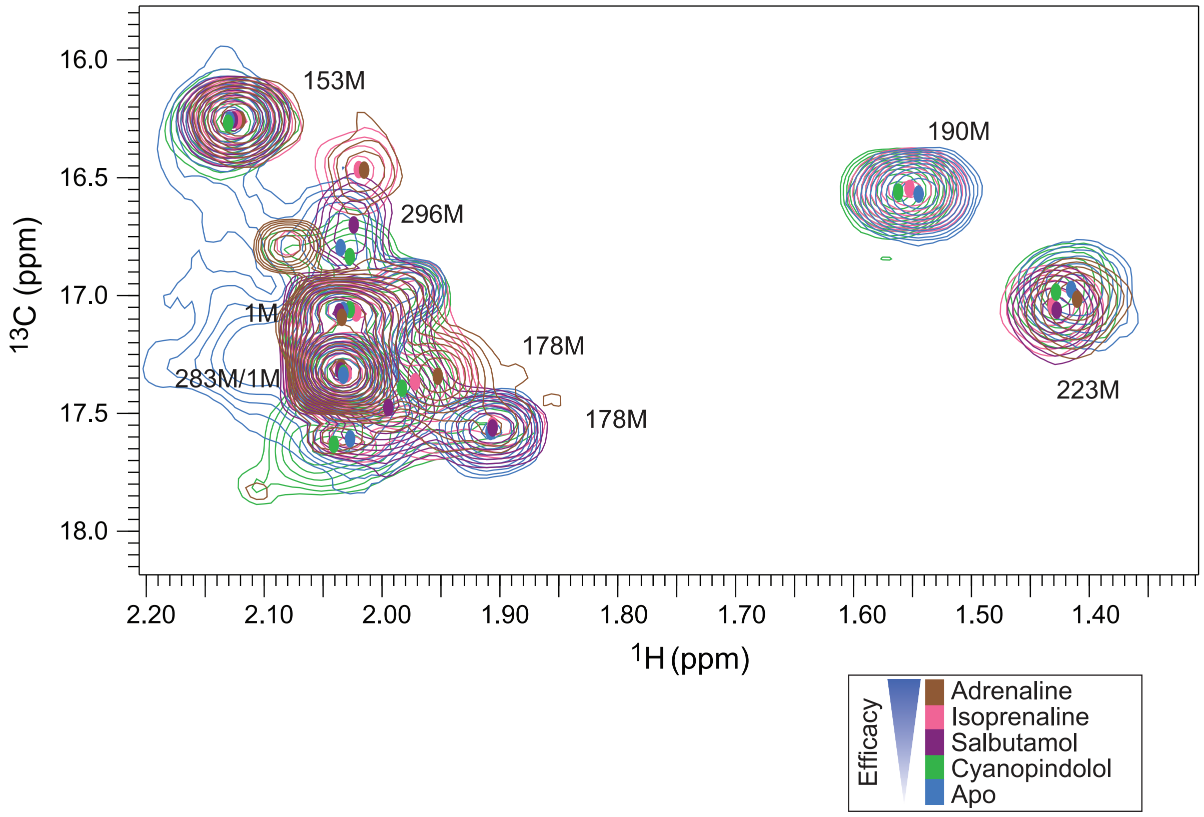

Supplementary Figure 9: Agonist-bound ternary complexes reveal ligand-dependent structural differences.

Methyl region of 2D ^{1}H,^{13}C HMQC spectra for [^{13}C-methyl-Met] β_1AR-MetΔ5 or β_1AR-MetΔ5-L190M bound to Nb6B9 in the apo form (blue) or in complex with different orthosteric agonists; cyanopindolol (weak partial agonist, green), salbutamol (partial agonist, purple), isoprenaline (full agonist, pink) and adrenaline (full agonist, brown). Due to limited sample availability two β_1AR constructs were used: β_1AR-MetΔ5-L190M was used with isoprenaline, cyanopindolol and in the apo form, while β_1AR-MetΔ5 was used with salbutamol and adrenaline. Except for the absence of the M190 signal in the spectra of the latter, the two β_1AR constructs were identical in terms of stability and binding properties and hence were used interchangeably.

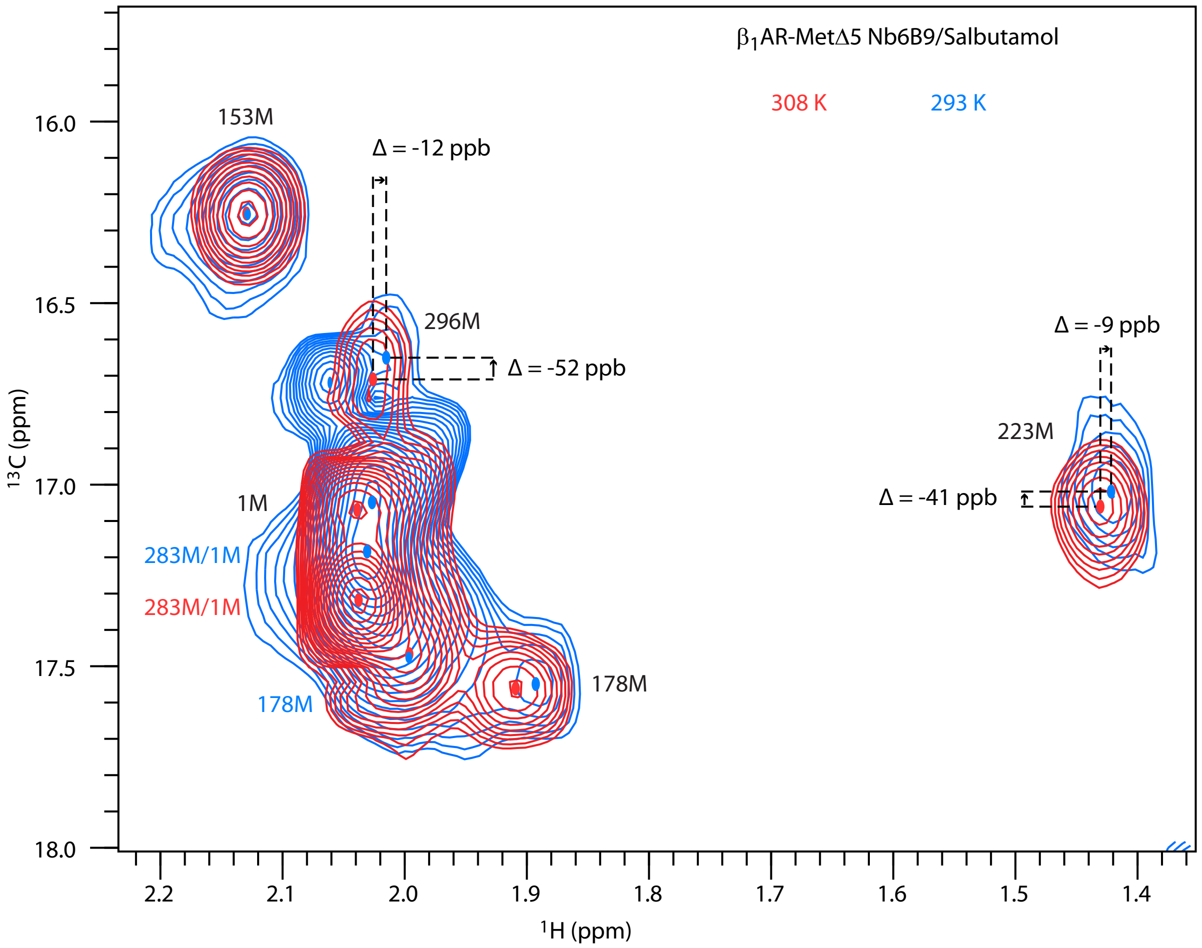

Supplementary Figure 10: Temperature-dependent chemical shift changes of the M223$^{5.54}$ and M296$^{6.41}$ resonances indicate a fast-exchange equilibrium in the ternary complex of β_1AR-MetΔ5 bound to salbutamol and Nb6B9. Chemical shift changes towards smaller values are observed for the M223$^{5.54}$ and M296$^{6.41}$ resonances between the spectra recorded at 308 K and 293 K (see Supplementary Fig. 7). The temperature dependence suggests a fast-exchange equilibrium, with a shift away from the conformationally less constrained state as the temperature is lowered (see Supplementary Note 3). Lowering the temperature also leads to a decrease in relative peak intensities suggesting a slight shift from faster exchange towards the intermediate exchange regime.

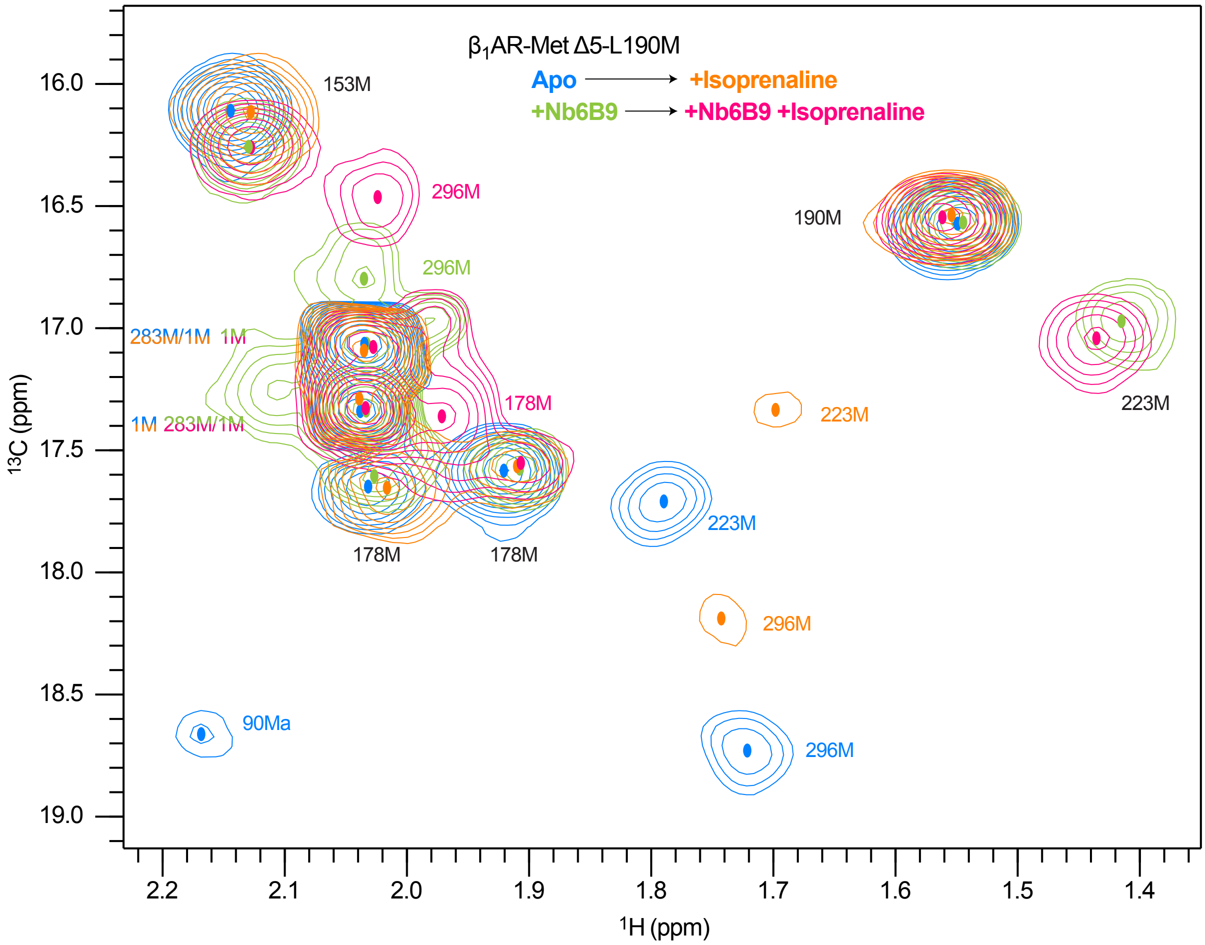

Supplementary Figure 11: The basal complex with nanobody shows similarities to the ternary receptor complex with agonist and nanobody. Methyl region of 2D ^{1}H,^{13}C HMQC spectra for [^{13}C-methyl-Met] β_1AR-MetΔ5-L190M comparing the chemical shift changes between the apo (blue) and isoprenaline-bound receptor (orange) and the ligand-free basal active complex bound to Nb6B9 alone (green) and ternary complex with Nb6B9 and isoprenaline-bound (pink). The similarity in resonance positions suggests that the basal active complex is structurally closer to the ternary complex than to the apo or isoprenaline-bound receptor.

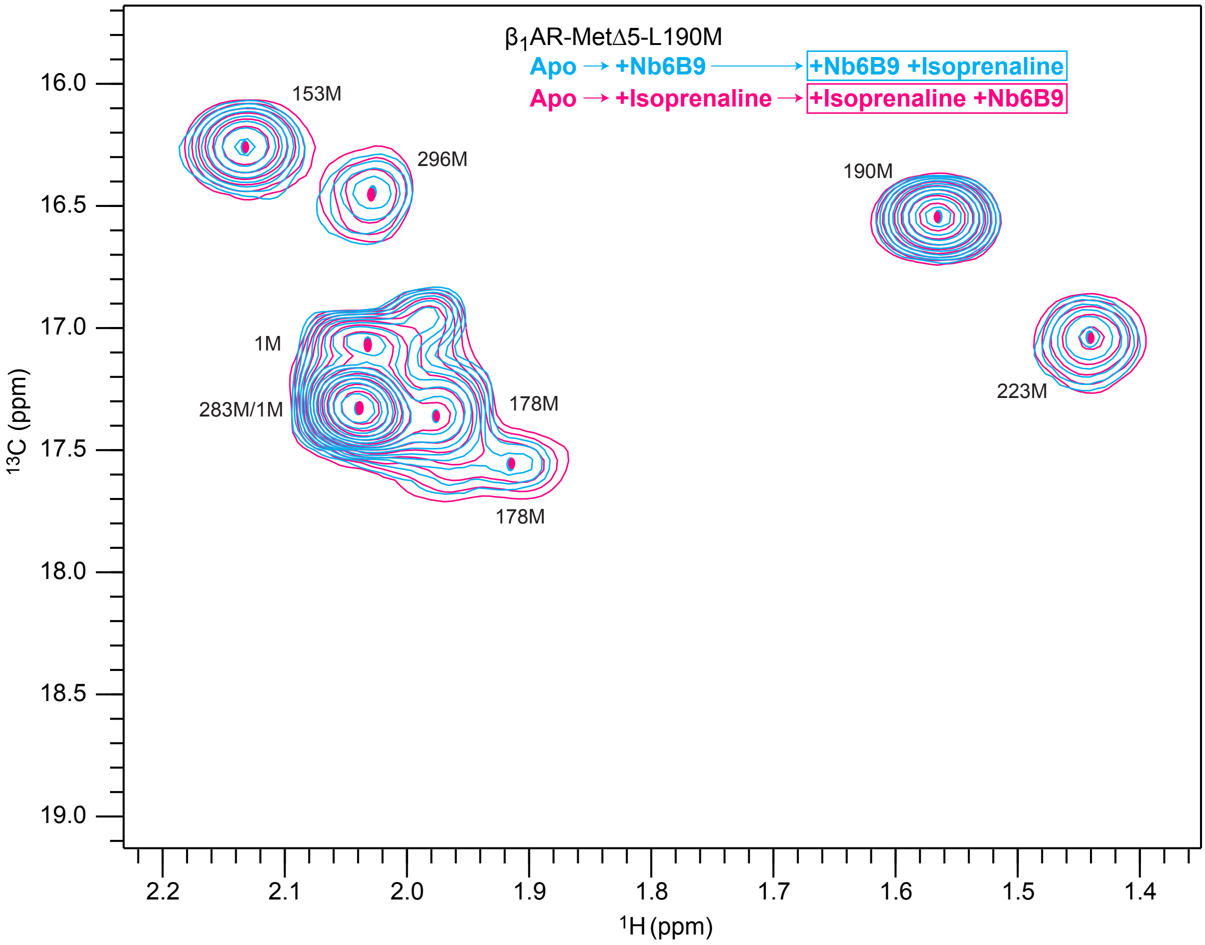

Supplementary Figure 12: Ternary complex formation does not depend on the order of binding. Methyl region of 2D ^{1}H,^{13}C HMQC spectra for [^{13}C-methyl-Met] β_1AR-MetΔ5-L190M shows that identical spectra for the isoprenaline-bound ternary complex are obtained regardless of whether ligand or nanobody is added to the receptor first. In blue, apo form was bound to Nb6B9 to form the basal active complex before addition of isoprenaline. In pink, isoprenaline was first added to the apo form, followed by Nb6B9. The spectra displayed are for the ternary complexes in each case, as indicated by the coloured rectangles in the key.

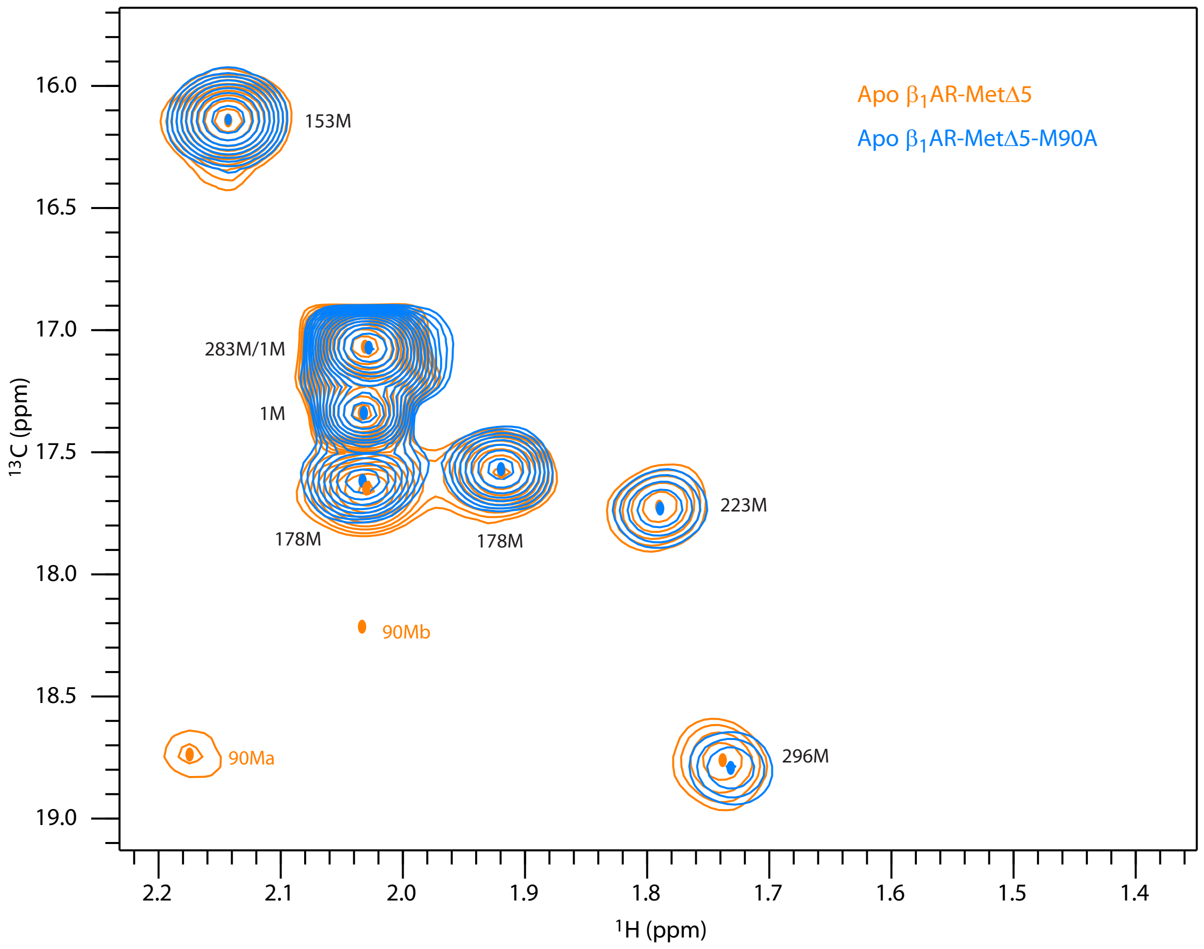

Supplementary Figure 13: Thermostabilising Mutation M90A.
Methyl region of 2D ^{1}H,^{13}C HMQC spectra for [^{13}C-methyl-Met] β_1AR-MetΔ5 (orange) and β_1AR-MetΔ5-M90A (blue) indicate little change due to the additional thermostabilising mutation M90A. This is in contrast to the ligand-bound receptor and the ternary complexes (see Fig. 7).

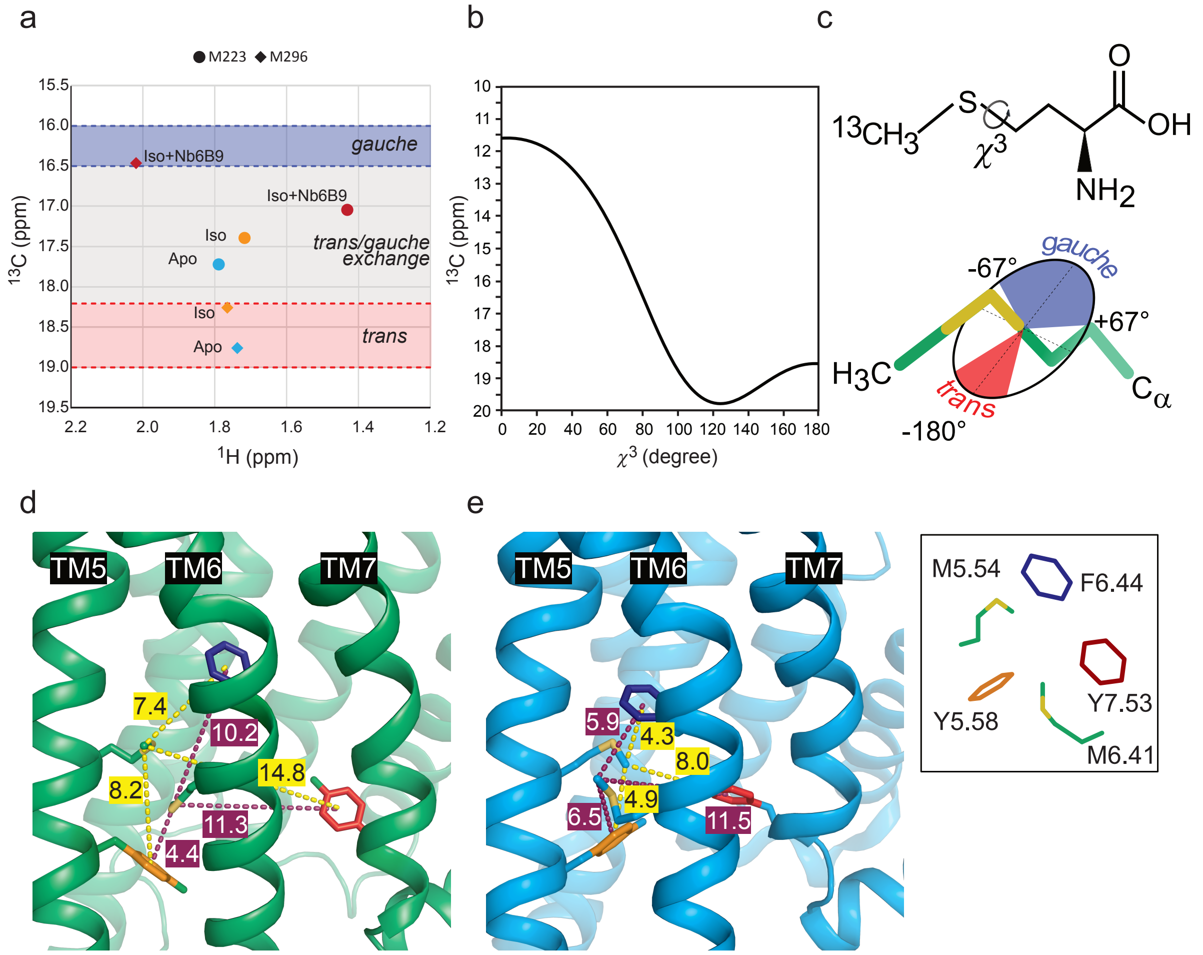

Supplementary Figure 14: Methyl-methionine χ^3 rotamer conformations and local environment of M5.54 and M6.41.
Methyl ^{13}C chemical shift positions are a sensitive reporter of χ^3 rotamer conformations, while ^{1}H shift positions are strongly influenced by the ring current shifts of neighbouring aromatic residues. (a) shows a schematic [^{1}H,^{13}C] correlation spectrum, with the chemical shifts for M223$^{5.54}$ and M296$^{6.41}$ in various states indicated along with approximate ^{13}C chemical shift regions for trans, trans/gauche exchanging and gauche rotamer positions: β_1AR in the apo form (blue), isoprenaline-bound β_1AR (orange), β_1AR/Isoprenaline/Nb6B9 ternary complex (red). (b) shows a simulation of the variation in ^{13}C chemical shift with χ^3 rotamer conformations taken from Butterfoss et al.[7] (c) shows the various χ^3 rotamer positions in the context of the methionine side chain, with the preferred regions highlighted. (d) and (e) show the inverse agonist-(carazolol) bound β_2AR (2RH1) (d) and the full-agonist (BI-167107), G_s-bound β_2AR (3SN6). The aromatic side chains, which can influence the ^{1}H shifts of M215$^{5.54}$

(equivalent to M223$^{5.54}$ in our study) and M279$^{6.41}$ (equivalent to M296$^{6.41}$ in our study) via ring current effects, are highlighted with Tyr$^{5.58}$ in orange, Phe$^{6.44}$ in blue and Tyr$^{7.53}$ in red. β_2AR structures were used since in the available β_1AR structures, Y227$^{5.58}$ is mutated to an alanine, whereas the β_1AR construct used in our study contained Y227$^{5.58}$. Distances are marked on the structure in angstroms with measurements from M223$^{5.54}$ shown in yellow, while measurements from M296$^{6.41}$ are indicated in purple. In (e), the χ^3 conformation for Met$^{6.41}$ was adjusted to the predicted gauche conformation ($-67°$) based on our NMR data, in contrast to the value of $-151.65°$ in the β_2AR structure. Activation results in an increase in the gauche conformation for Met$^{5.54}$ and Met$^{6.41}$. A substantial displacement of Tyr$^{7.53}$ towards TM6 can be observed in the activated β_2AR structure (d), along with alteration in the χ^1 rotamer conformation of Tyr$^{5.58}$ and movement of Phe$^{6.44}$ towards Met$^{5.54}$ resulting in an upfield shift of M223$^{5.54}$. Tyr$^{5.58}$ and Phe$^{6.44}$ also move closer to Met$^{6.41}$ and along with the change in χ^3 rotamer conformation for Met$^{6.41}$ result in deshielding (downfield shift) of M296$^{6.41}$.

Supplementary Table 1

Sequence alignments for methionine residues studied in this paper with other class A GPCRs

Methionine position in turkey β_1AR-MetΔ5 construct	M90	L108M	M153	M178	L190M	M223	M283	M296
Ballesteros-Weinstein numbering	2.53	EL1	IL2	4.62	EL2	5.54	6.28	6.41
Human β1‡	M107	E125	L170	L195	R207	M240	L317	M330
Human β2‡	M82	T100	L145	Q170	I182	M215	L266	M279
Human β3‡	M86	P104	V149	M174	A186	M220	L285	M298
Human A_{2A}‡	V82	Y100	R145	I170	P181	M212	N367	I380
Most common residue (%)§	V 29.5%	P 18%#	R 31%#	L 19.15%	$	I 33.21%	K 18.73%	V 38.53%
% of class A receptors with Met at this site§	12.74%	1%#	6%#	3.65%	$	32.43%	1.38%	13.64%
Rank of Met in order of most common residues§	3rd	18th #	3rd #	7th	$	2nd	17th	3rd
Mutation for assignment	A	L	A	A	L	L	A	A

‡Information obtained using GPCRdb residue tables (http://gpcrdb.org/residue/residuetable) except for loop residues where the GPCRdb structure-based alignment tool was used (http://gpcrdb.org/alignment/targetselection)
§Except for L108 and M153, all information from GMOS web interface (http://lmc.uab.cat/gmos/)
#Using GPCRdb structure-based alignment tool (http://gpcrdb.org/alignment/targetselection) with all class A GPCRs selected.
$Alignment could not be performed for EL2

Supplementary Table 2

Pharmacological properties for ligands used in this study

Ligand[#]	Abbreviation	Classification	Efficacy, ^{3}H-cAMP accumulation assays in CHO (% ISO activity)[‡§]	pK_D from ^{3}H-CGP12177 whole cell binding studies in CHO cells[†§]
**7-Methylcyanopindolol**	7mC	Very weak partial agonist	2.3±0.3[§]	10.37±0.03[§]
**Carvedilol**	CAR	Weak partial agonist	12±0.4	9.43±0.05
**Cyanopindolol**	CYP	Weak partial agonist	39±1	10.89±0.06
**Salbutamol**	SAL	Partial agonist	97±2	4.99±0.03
**Isoprenaline**	ISO	Full agonist	100	6.86±0.08
**Adrenaline**	ADR	Full agonist	110±2	6.01±0.04

‡Data taken from Baker et al. 2011.[15]
†Data taken from Baker et al. 2010.[16]
§The values for 7-methylcyanopindolol are taken from Sato et al. 2015.[17]
#Molecular structures of the ligands used in this study:

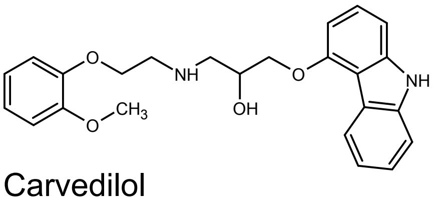

Carvedilol

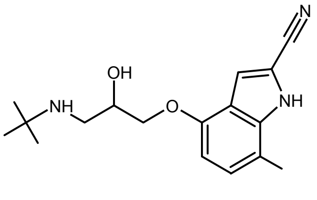

7-Methylcyanopindolol

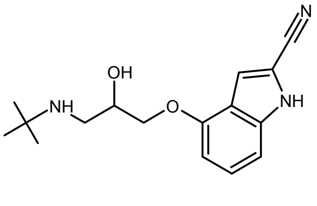

Cyanopindolol

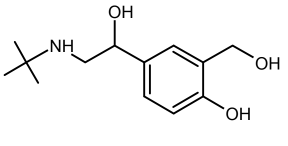

Salbutamol

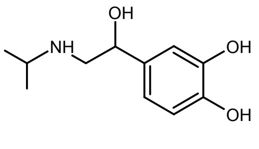

Isoprenaline

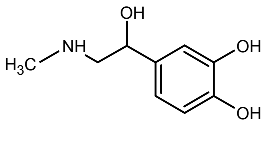

Adrenaline

Supplementary Table 3

Chemical exchange rates estimated from chemical shift differences for equilibria studied

Equilibrium	Construct	Spectra compared	Temperature	Δω /Hz		NMR timescale	k_{ex} estimate /Hz
				**M223**	**M296**		
**Ligand exchange** $\mathbf{I \rightleftharpoons A}$	MetΔ5	Apo, +ISO	308 K	87.8	103.1	Fast	> 100
**Nanobody binding**	MetΔ5<br>MetΔ5-L190M	+ISO,<br>+ISO+Nb6B9	308 K	239.4	414.4	Slow	< 230
**Ternary complex equilibrium** $\mathbf{A^{G-} \rightleftharpoons A^{G+}}$	MetΔ5-L190M	Apo + Nb6B9, Iso+Nb6B9	308 K	19.5	67.8	Fast	> 68
**Pre-active state equilibrium** $\mathbf{A' \rightleftharpoons A''}$	MetΔ5-L190M	+ISO (see Figure 3)	298 K	48.7		Slow	< 49

Supplementary Table 4

a) Relative peak intensity of methyl resonances M223 and M296 for β$_1$AR-MetΔ5-L190M, β$_1$AR-MetΔ5, β$_1$AR-MetΔ5-M178A and β$_1$AR-MetΔ5-M283A[#]

	β$_1$AR							
	MetΔ5-L190M		MetΔ5		MetΔ5-M178A		MetΔ5-M283A	
Ligand	M223	M296	M223	M296	M223	M296	M223	M296
Adrenaline			0.06	0.04				
Isoprenaline	0.08	0.12	0.05	0.10	0.04	0.10	0.04	0.08
Salbutamol			0.16	0.22			0.12	0.18
Cyanopindolol	0.28	0.24	0.25	0.23				
7-Methylcyanopindolol			0.27	0.21			0.28	0.23
Apo	0.26	0.21	0.27	0.22	0.25	0.20	0.26	0.27
Carvedilol			0.27	0.25				
Apo/Nb80	0.38	0.36	0.44	0.29	0.54[$]	0.28	0.47	0.27
Apo/Nb6B9	0.43	0.36						
Adr/Nb6B9			0.60	0.35				
Iso/Nb80	0.57	0.39	0.59	0.35	0.50	0.39	0.50	0.33
Iso/Nb6B9	0.57	0.39						
Sal/Nb6B9			0.59	0.36				
Cyp/Nb6B9	0.47	0.37						

[#]All signal intensities are relative to the intensity of M153 within the same spectrum. All data was extracted from ^{13}C HMQC spectra recorded at 800 MHz (^{1}H) and 308 K. Lower values correspond to increased levels of dynamics on the µs-to-ms timescale. A graphical representation of the data for β$_1$AR-MetΔ5 and β$_1$AR-MetΔ5-L190M is shown in Supplementary Fig. 4a and b.

[$]Measurement was affected by signal overlap.

b) Normalised peak intensity of methyl resonances M223 and M296 for β_1AR-MetΔ5-L190M, β_1AR-MetΔ5, β_1AR-MetΔ5-M178A and β_1AR-MetΔ5-M283A#

	β_1AR							
	MetΔ5-L190M		MetΔ5		MetΔ5-M178A		MetΔ5-M283A	
Ligand	M223	M296	M223	M296	M223	M296	M223	M296
Adrenaline			0.1	0.11				
Isoprenaline	0.14	0.31	0.08	0.28	0.07	0.26	0.08	0.24
Salbutamol			0.27	0.61			0.24	0.55
Cyanopindolol	0.49	0.62	0.42	0.64				
7-Methylcyanopindolol			0.45	0.58			0.56	0.7
Apo	0.46	0.54	0.45	0.61	0.46	0.51	0.52	0.82
Carvedilol			0.45	0.69				
Apo/Nb80	0.67	0.92	0.73	0.81	1	0.72	0.94	0.82
Apo/Nb6B9	0.75	0.92						
Adr/Nb6B9			1	0.97				
Iso/Nb80	1	1	0.98	0.97	0.93	1	1	1
Iso/Nb6B9	1	1						
Sal/Nb6B9			0.98	1				
Cyp/Nb6B9	0.82	0.95						

#The normalisation is based on the relative intensity data shown in Supplementary Table 4a. Accordingly, the most intense signal within the ligand series of a particular receptor construct is given a normalised intensity value of one. The signals for M223 and M296 are normalised individually. Lower values correspond to increased levels of dynamics on the µs-to-ms timescale. A graphical representation of the data is shown in Fig. 2a,b and Supplementary Fig. 6.

Supplementary Table 5

Normalised signal intensity of methyl resonances M223 and M296 of β$_1$AR-MetΔ5-L190M relative to different reference signals#

	β$_1$AR-MetΔ5-L190M					
	relative to M153		relative to M190		relative to LMNG (CH_3)	
**Ligand**	**M223**	**M296**	**M223**	**M296**	**M223**	**M296**
Isoprenaline	0.14	0.31	0.15	0.29	0.16	0.30
Cyanopindolol	0.49	0.62	0.51	0.52	0.47	0.65
Apo	0.46	0.54	0.45	0.55	0.47	0.50
Apo/Nb80	0.67	0.92	0.69	0.83	0.63	0.97
Apo/Nb6B9	0.75	0.92	0.72	0.88	0.77	0.95
Iso/Nb80	1.00	1.00	0.98	1.00	0.97	0.95
Iso/Nb6B9	1.00	1.00	1.00	0.90	1.00	1.00
Cyp/Nb6B9	0.82	0.95	0.80	0.83	0.77	0.92

#The same intensity data for M223 and M296 was normalised using the signals of M153, M190 and the methyl group signal of receptor-bound LMNG (Supplementary Fig. 5b) as the reference. The data shows that the normalised intensities are very similar for any of the reference signals. All signal intensities were extracted from ^{13}C HMQC spectra recorded at 800 MHz (^1H) and 308 K.

Supplementary Table 6

Temperature dependence of relative signal intensity and signal-to-noise ratio (SNR) for the M223 and M296 methyl groups for the ternary complex of β_1AR-MetΔ5 with salbutamol and Nb6B9 reveal the presence of exchange contributions

	β_1AR-MetΔ5 with salbutamol / Nb6B9							
	relative intensity (reference M153) #		signal-to-noise ratio (SNR) †					
			uncorrected			corrected for T and η (back calculated to 308 K)		
Temperature	M223	M296	M223	M296	M153 (ref)	M223	M296	M153 (ref)
308 K	0.59	0.36	130	80	219	130	80	219
293 K	0.46	0.29	64	45	145	94	65	212
ratio 293 K vs 308 K	0.78	0.81				0.72	0.81	0.97

#For each of the spectra relative signal intensities were measured against the signal of M153 as the reference.

†Signal-to-noise ratios (SNR) are shown both in uncorrected and corrected form. The correction is applied to back-calculate the values measured at 293 K to 308 K i.e. to compensate for the differences in temperature and viscosities enabling a direct comparison of the values measured at the two temperatures. Clearly, this is an approximation as other processes can affect the signal intensity. Compensating for the temperature and viscosity differences the SNR values at 293 K were multiplied by 1.465 i.e. $(\eta_{308}/\eta_{293})*(308/293)$, with η_{293} = 1.002 cP, η_{308} = 0.7192 cP. (Viscosity values of pure water were used which is an approximation, as effects due to the presence of protein and detergent were ignored.)

In the absence of any exchange contributions the SNR value measured at 293 K and back-calculated to 308 K should be very similar to the value measured at 308 K. Hence the ratio of the values at 293 K and 308 K is close to one. This can be seen for M153, which does not have any µs-to-ms exchange contributions over this temperature range. In contrast, back calculated values for M223 and M296 are smaller than the ones directly measured at 308 K. This indicates the presence of increased exchange contributions for M223 and M296 at the lower temperature, which reduce the SNR of these signals. As explained in the main text, this is in agreement with an equilibrium that at 308 K is in fast exchange on the NMR timescale and where, as the temperature is lowered, exchange becomes more prominent.

Supplementary Table 7

a) Temperature and field strength dependence of relative signal intensities of methyl resonances M223 and M296 of β$_1$AR-MetΔ5-M90A[#]

	β$_1$AR-MetΔ5-M90A Isoprenaline	
	relative intensity	
**Temperature / ^{1}H**	**M223**	**M296**
308 K (800 MHz)	0.35	0.31
293 K (800 MHz)	0.34	0.29
800 MHz (308 K)	0.34	0.29
600 MHz (308 K)	0.34	0.28

[#]Relative intensities are measured against the corresponding M153 signal for each spectrum at different temperatures (293 K and 308 K, both at 800 MHz (^{1}H)) and different field strengths (600 and 800 MHz ^{1}H frequency at 308 K). The relative signal intensities show no indication of any field or temperature dependence.

b) Ternary complexes of β$_1$AR-MetΔ5 and β$_1$AR-MetΔ5-M90A: Comparison of relative signal intensities for the methyl resonances of M223 and M296[$]

	β$_1$AR			
	MetΔ5		MetΔ5-M90A	
**Ligand**	**M223**	**M296**	**M223**	**M296**
Iso/Nb80	0.54[#]	0.35[#]	0.57	0.37
Iso/Nb6B9	0.57[†]	0.39[†]	0.52	0.29
Sal/Nb6B9	0.59[§]	0.36[§]		
Cyp/Nb6B9	0.47[‡]	0.37[‡]	0.48	0.28

[#]Intensities represent average values of measurements for β$_1$AR-MetΔ5, β$_1$AR-MetΔ5-L108M, β$_1$AR-MetΔ5-M178M, β$_1$AR-MetΔ5-M178M, β$_1$AR-MetΔ5-L190M
[†]Measured on β$_1$AR-MetΔ5-L190M
[§]Measured on β$_1$AR-MetΔ5
[‡]Measured on β$_1$AR-MetΔ5-L190M
[$]All signal intensities were extracted from ^{13}C HMQC spectra recorded at 800 MHz (^{1}H) and 308 K

Supplementary Table 8

Comparison of methionine χ^3 conformations: M223$^{5.54}$ and M296$^{6.41}$ of β$_1$AR and M215$^{5.54}$ and M279$^{6.41}$ of β$_2$AR

			X-ray							NMR			
GPCR	Ligand	Pharmacology‡	PDB ID $	mutation	conformation	M223$^{5.54}$		M296$^{6.41}$		M223$^{5.54}$		M296$^{6.41}$	
				Tyr$^{5.58}$, Tyr$^{7.53}$	Cytoplasm#	χ^3 rotamer (˚)		χ^3 rotamer (˚)		^{13}C (ppm)	χ^3 rotamer §	^{13}C (ppm)	χ^3 rotamer
β$_1$AR	bucindolol	partial agonist	4AMI	Y227A	inactive	66.6	*gauche*	-164.7	*trans*				
β$_1$AR	(S)-Cyanopindolol	weak p.a.	2VT4	Y227A	inactive	69.0	*gauche*	-167.2	*trans*				
β$_1$AR	Carmoterol	full agonist	2Y02	Y227A	inactive	76.0	*gauche*	-171.6	*trans*				
β$_1$AR	(S)-Cyanopindolol	weak p.a.	5F8U	Y227A	inactive	67.6	*gauche*	-161.1	*trans*				
β$_1$AR	(S)-Cyanopindolol	weak p.a.	2YCX	Y227A	inactive	83.1	*gauche*	-168.1	*trans*				
β$_1$AR	timolol	antagonist	2YCZ	Y227A	inactive	73.1	*gauche*	-178.8	*trans*				
β$_1$AR	7-Methylcyanopindolol	very weak p.a.	5A8E	Y227A,Y343L	inactive	70.6	*gauche*	-168.4	*trans*				
β$_1$AR	(S)-Cyanopindolol	weak p.a.	4BVN	Y227A,Y343L	inactive	77.9	*gauche*	-165.2	*trans*				
β$_1$AR	(S)-Cyanopindolol	weak p.a.	2YCY	Y227A	inactive	67.9	*gauche*	-168.0	*trans*	17.60	*gauche/trans*	18.63	*trans*
β$_1$AR	Isoprenaline	full agonist	2Y03	Y227A	inactive	72.6	*gauche*	178.2	*trans*	17.33	*gauche/trans*	18.20	*trans*
β$_1$AR	Apo	no ligand			inactive					17.67	*gauche/trans*	18.70	*trans*
β$_1$AR	Nb6B9	basal complex			active					16.97	*gauche/(trans)*	16.79	*gauche*
β$_1$AR	Cyp/Nb6B9	p.a./ternary			active					16.98	*gauche/(trans)*	16.83	*gauche*
β$_1$AR	Iso/Nb6B9	full a./ternary			active					17.04	*gauche/(trans)*	16.46	*gauche*
						M215$^{5.54}$		M279$^{6.41}$		M215$^{5.54}$		M279$^{6.41}$	
β$_2$AR	BI-167107/Nb6B9	full a./ternary	3SN6		active	85.4	*gauche*	-138.4	~*trans*	17.3	*gauche/trans*	16.7	*gauche*
β$_2$AR	BI-167107/G$_s$	full a./ternary	3P0G		active	58.9	*gauche*	-151.7	~*trans*				

$Selected β$_1$AR structures where B factor of the methyl groups of M223 and M296 is smaller than the mean B factor of the overall structure.

#Inactive/active classification according to the conformational arrangement of the cytoplasmic side of TM6.

§gauche/trans: fast exchange between gauche and trans χ^3 rotamers; gauche/(trans): is representative of a fast exchange with a larger gauche population.

‡a., agonist; p.a., partial agonist

Supplementary Table 9

Comparison of methyl group environments: Distance to proximal aromatic residues and ^{1}H chemical shifts of $Met^{5.54}$ and $Met^{6.41}$

			Inactive receptor	Active receptor
Complex			β_2AR carazolol	β_2AR BI-167107/G_s
PDB ID			2RH1	3SN6
Pharmacology			inverse agonist	full agonist/ternary
Distance (Å) (pseudo atom of CH_3 group to center of aromatic ring)	$Met^{5.54}$	$Tyr^{5.58}$	8.2	4.9
	$Met^{5.54}$	$Phe^{6.44}$	7.4	4.3
	$Met^{5.54}$	$Tyr^{7.53}$	14.8	8
	$Met^{6.41}$	$Tyr^{5.58}$	4.4	6.5
	$Met^{6.41}$	$Phe^{6.44}$	10.2	5.9
	$Met^{6.41}$	$Tyr^{7.53}$	14.8	11.5
^{1}H chemical shift (ppm)	Experimental#	$M223^{5.54}$	1.78	1.43
	Experimental#	$M296^{6.41}$	1.72	2.02
	Calculated$	$M215^{5.54}$	2.13	1.55
	Calculated$	$M279^{6.41}$	1.68	2.27

#Measured on β_1AR in the apo form, representative of the inactive receptor, and β_1AR bound to isoprenaline and Nb6B9 for the active receptor.

$Chemical shifts for methyl group Hε were calculated based on the Johnson-Bovey equation[10] using the crystallographic coordinates. Individual methyl proton contributions were averaged as pseudo-atom.

Supplementary Table 10

Primer sequences used for site-directed mutagenesis

**M44L**	Forward	GGGAGGCGGGCCTGAGCCTGCTG
	Reverse	CAGCAGGCTCAGGCCCGCCTCCC
**M48L**	Forward	GCCTGCTGCTGGCCCTGGTGGTGC
	Reverse	GCACCACCAGGGCCAGCAGCAGGC
**M179L**	Forward	CCTGCCCATCATGCTGCACTGGTGGCGGG
	Reverse	CCCGCCACCAGTGCAGCATGATGGGCAGG
**M281A**	Forward	GACGTCCCGTGTCGCCGCCATGAGGGAACAC
	Reverse	GTGTTCCCTCATGGCGGCGACACGGGACGTC
**M338A**	Forward	CGCCAACTCTGCTGCCAACCCCATCATC
	Reverse	GATGATGGGGTTGGCAGCAGAGTTGGCG
**L190M**	Forward	CGAGGACCCTCAGGCGATGAAGTGCTACCAGG
	Reverse	CCTGGTAGCACTTCATCGCCTGAGGGTCCTCG
**L108M**	Forward	GCACCTGGATGTGGGGCTCC
	Reverse	GGAGCCCCACATCCAGGTGC
**M90A**	Forward	GCGCCGACCTGGTGTACGGGCTGCTGGTGG
	Reverse	CCACCAGCAGCCCGTACACCAGGTCGGCGC
**M153A**	Forward	GCTACCAGAGCCTGGCTACCAGGGCTCGGG
	Reverse	GGCCCGAGCCCTGGTAGCCAGGCTCTGGTAGC
**M178A**	Forward	CCTGCCCATCTACCTGCACTGGTGGCGGG
	Reverse	CCCGCCACCAGTGCAGGTAGATGGGCAGG
**M283A**	Forward	CGTCCCGTGTCGCCGCCTACAGGGAACACAAAGC
	Reverse	GCTTTGTGTTCCCTGTAGGCGGCGACACGGGACG
**M223L**	Forward	CATCCCCCTCCTCATCTACATCTTCGTGTACC
	Reverse	GGTACACGAAGATGTAGATGAGGAGGGGGATG
**M296A**	Forward	CATTGGGTATCATCTACGGGGTGTTCACCC
	Reverse	GGGTGAACACCCCGTAGATGATACCCAATG

Supplementary Table 11

Ligand affinities and bound populations for NMR samples used in this study

Ligand	K_D from ^{3}H-CGP12177 whole cell binding studies in CHO cells[†§]	Ligand-bound population[‡]
**7-Methylcyanopindolol**	42.66 pM[§]	>99.999%
**Carvedilol**	0.37 nM	>99.999%
**Cyanopindolol**	12.88 pM	>99.999%
**Salbutamol**	10.23 µM	>99.901%
**Isoprenaline**	138.04 nM	>99.988%
**Adrenaline**	0.98 µM	>99.969%

[†]Data taken from Baker et al. 2010.[16]
[§]The value for 7-methylcyanopindolol is taken from Sato et al. 2015.[17]
[‡]Ligand-bound population calculated using the sample protein concentration, ligand concentration added and the affinity.

Supplementary Note 1: NMR timescale

In solution NMR, two resonances in equilibrium may manifest in different ways in the NMR spectrum depending on the rate of exchange relative to the chemical shift difference between the resonances. If the exchange rate constant, k, is less than the chemical shift difference (expressed in Hz, ω) i.e. $k \ll \Delta\omega$, the two resonances will appear as two separate peaks in the NMR spectrum, i.e. the exchange process is sufficiently slow that both peaks can be resolved; this regime is known as *slow exchange*. In contrast, where $k \gg \Delta\omega$, it is not possible to resolve the two individual peaks and instead a single peak is observed at a position corresponding to a weighted average of the populations of the two contributing resonances. As the populations change e.g. due to temperature changes or addition of ligands which alter the populations of the two states in equilibrium, the position of this single peak will shift. This is known as *fast exchange* and is observed for residues e.g. M223$^{5.54}$ and M296$^{6.41}$ in the different ligand-bound states in this study. Between these regimes is the intermediate exchange timescale; in this region, peak broadening is observed leading to a loss of intensity and resolution. The comparison between the exchange rate and chemical shift difference as described here is known as the NMR timescale.

In the HMQC experiments used in this study to maximise the sensitivity of the methyl signals due to the favourable methyl-TROSY effect,[1] the presence of multiple quantum magnetisation in the ^{13}C evolution period complicates the comparison between the chemical shift difference and the exchange rate. We estimate this using the following formula:

$$\Delta\omega = \sqrt{(\delta H_a - \delta H_b)^2 + (\delta C_a - \delta C_b)^2} \tag{1}$$

where δH_a, δH_b, δC_a and δC_b represent frequencies (in Hz) in the proton (^{1}H) and carbon (^{13}C) dimensions respectively, for two different peaks, a and b. We identify four equilibria in our study with different exchange rates on the NMR timescale: ligand-exchange (fast exchange), nanobody binding (slow exchange), ternary complex equilibrium (fast exchange) and the pre-active state equilibrium (slow exchange). We compare frequencies for apo- and isoprenaline-bound forms, representing the ligand exchange equilibrium; isoprenaline-bound and isoprenaline + nanobody-bound to represent the nanobody-binding equilibrium; apo + nanobody and isoprenaline-bound + nanobody to represent the ternary complex equilibrium ($A^{G-} \rightleftharpoons A^{G+}$), and M223a and M223b peaks from the isoprenaline-bound form (Fig. 3) at 298 K representing slow exchange of the pre-active (A) state. In each case, based on identification of the equilibrium as slow or fast as discussed in the text, we provide a lower or upper bound for the exchange rate constant (k_{ex}). Data are shown in Supplementary Table 3.

Supplementary Note 2: Intensity comparisons as a proxy to assess conformational dynamics in β_1AR

GPCRs are highly dynamic molecules that show varying amounts of motions taking place on a range of timescales. As many of the residues in GPCRs are strongly broadened in NMR spectra and changes in linewidth vary with the activity state of the receptor, the presence of considerable amounts of µs-to-ms motions is suspected, presumably as a consequence of the extensive receptor plasticity. Many of the conformational dynamics, particularly those on the µs-to-ms timescale, are believed to be functionally relevant and reflect the signalling characteristics of the receptor state. Hence it is important to characterise the dynamics and enable comparisons between the different receptor states. NMR spectroscopy typically uses spin relaxation techniques for quantitative or qualitative descriptions of protein dynamics. Such approaches are often limited by overall sensitivity and in view of the extended measurement times require long sample stability. Our NMR measurements were restricted by relatively low protein concentrations and limited sample stability (due to the use of a construct with reduced thermostabilisation). The size of the protein-detergent complex (~80 kDa) was also challenging but within the range of feasibility for methyl spectroscopy methods. However, the use of non-deuterated β_1AR samples substantially reduced the performance of TROSY relaxation-compensation in the ^{13}C HMQC experiments,[1] due to unfavourable inter-proton interactions and made the use of relaxation techniques, in particular CPMG-based methods, very difficult due to rapid signal decay and inherently low sensitivity. In the presence of conformational dynamics that result in additional line broadening, further dramatic sensitivity losses had to be expected precluding the use of such measurements in our β_1AR study.

Information on slower µs-to-ms dynamics that result in further line broadening can also be obtained from signal linewidths. Our NMR data, however, was recorded to maximize the signal-to-noise ratio and hence the resolution in the indirect ^{13}C dimension was kept low, making it difficult to extract spin lifetime information of sufficient quality from the truncated data. Accordingly, we made use of intensity information as a proxy to assess changes in methionine methyl group dynamics taking place on a slower timescale. Intensity ratios enabled a semi-quantitative evaluation of receptor dynamics and how these change depending on the receptor states. In this work substantial intensity variations were interpreted as resulting from changes in µs-to-ms dynamics of methyl groups and their local environment. Methyl groups are typically known to have very favourable relaxation properties where rapid rotation around the methyl group symmetry axis results in long T_2 values and relatively low S^2 order parameters (indicative of motion decoupled from the overall global tumbling of the protein molecule). The resulting sharp signals make methyl detected experiments a

powerful NMR method to study macromolecules.[1,2] The relatively long spin lifetimes also make methyl groups very sensitive for the detection of changes in dynamics on slower timescales, including motions on the µs-to-ms timescale related to conformational exchange processes, which typically lead to exchange broadened signals and a reduction in signal intensity as observed in this study. Frequently in the main text we refer to more mobile, more dynamic or less rigid receptor states, and such descriptions refer to a motional regime of the receptor that leads to additional signal broadening, which manifests itself experimentally through substantially reduced signal intensities. Exchange processes taking place on the µs-to-ms timescale are the cause for this. This motional regime has to be clearly separated from dynamics taking place on faster timescales, which typically result in the sharpening of signals either due to shorter internal correlation times or a reduction in motional order parameters when the entity becomes more dynamic. In the current work we were interested in monitoring differences in receptor characteristics accompanying ligand binding and the formation of complexes with nanobodies, with and without orthosteric ligands. Our main approach used relative and normalised signal intensities, both relating to the same spectroscopic data. Relative intensities allow the comparison of different methyl groups with each other as measured against a reference signal; the lower relative intensity reflecting a methyl group that is conformationally more dynamic on a timescale leading to signal broadening. Normalised intensities on the other hand offer a convenient way to compare different receptor constructs and samples. Intensity changes can be measured using sensitive ^{13}C HMQC experiments and enable the detection of exchange processes that contribute to line broadening. A discussion of changes in motional amplitudes or faster timescale motions based on our intensity measurements, however, is more difficult and will require the measurement of spin relaxation data. A more quantitative assessment of methyl relaxation for β_1AR in different receptor states is currently under way in our laboratory where we aim at the characterisation of motional timescales.

Supplementary Note 3: Conformational interpretation of ^{1}H and ^{13}C chemical shift data for M223$^{5.54}$ and M296$^{6.41}$ and interpretation of the observed temperature dependence

A substantial number of X-ray crystal structures of turkey β_1AR in ligand-only bound states have been determined (selected structures are listed in Supplementary Table 8). However all of these studies used thermostabilised receptor that contains the Y227A$^{5.58}$ mutation, which has been shown to impair coupling to IBPs e.g. nanobodies and G_s.[3] Some of these structures contain the additional thermostabilising mutation Y343L$^{7.53}$ that also prevents coupling. Notably a number of these structures bound to a series of different ligands (partial agonists salbutamol and dobutamine; full agonists carmoterol and isoprenaline) appear to be biased towards the inactive conformation, with the structures almost identical to antagonist-bound structures.[14] Changes concentrate in the ligand binding pocket with isoprenaline forming a hydrogen bond to Ser215$^{5.46}$ and a 1.0 Å, contraction of the binding pocked observed. However, changes on the cytoplasmic side of the receptor are not observed in these structures, with no outward movement of the cytoplasmic end of helix 6 and no substantial changes occurring to residues such as M223$^{5.54}$ and M296$^{6.41}$ studied in this paper.[12,14] In contrast the constructs used in our NMR study contain both Y227$^{5.58}$ and Y343$^{7.53}$ and use substantially reduced levels of thermostabilisation. Accordingly, they show competence in binding to IBPs. At the time of writing there are no active β_1AR structures coupled to IBPs available to compare our results against, however, a comparison can be made with active structures of human β_2AR.[4,5]

Methyl ^{13}C chemical shift information is a sensitive reporter of χ^3 rotamer conformation,[6,7] while the ^{1}H chemical shift information reports on the proximity and orientation of aromatic side chains to the methyl group protons due to the dominance of aromatic ring current shifts. Typical methyl methionine ^{13}C chemical shift values of $\sim 18.5 - 19$ ppm are representative of a χ^3 *trans* rotamer arrangement ($\chi^3 \sim \pm 180°$), while values of ~ 16 ppm are typical of a *gauche* arrangement ($\chi^3 \sim \pm 67°$). Values between the two, $\sim 17 - 17.8$ ppm indicate that multiple rotamers are sampled that are in fast exchange e.g. *trans*/*gauche* χ^3 rotamers (Supplementary Fig. 14a–c).

It would be of great value to compare the crystallographic information on M223$^{5.54}$ and M296$^{6.41}$ rotamer orientation with the NMR data in our study. However, due to the additional thermostabilisation that was required for the crystallisation of β_1AR, crystal structures bound to full agonist do not show the typical overall large-scale structural changes at the cytoplasm indicative of an activated receptor. It is generally assumed that in these crystal structures the cytoplasmic side of the receptor is maintained in a conformation characteristic of the inactive receptor, presumably as a consequence of

thermostabilisation that includes mutation of the key residues Y227$^{5.58}$ and Y343$^{7.53}$. While this prevents a direct comparison of active state β$_1$AR information, our data on inactive receptor states can be compared with the various crystal structures that present the cytoplasmic side of the receptor in an inactive state. It needs to be taken into account, however, that a comparison of the results of NMR and crystallographic studies can be limited due to the higher temperature used in the NMR investigations, which can result in conformational averaging in NMR rather than adopting a unique χ^3 conformation as seen in crystallography. This is particularly so for methionine residues where the energetic barrier for χ^3 rotation is quite low.[7]

Ligand-only bound states of β$_1$AR

^{13}C chemical shift data: Based on ^{13}C chemical shift arguments, all ligand-only bound states show M296$^{6.41}$ in a *trans* χ^3 rotamer orientation (18.2–18.7 ppm), while M223$^{5.54}$ (17.3–17.7 ppm) is sampling *gauche* and *trans* conformations, exchanging rapidly between the two rotamer orientations. Crystal structures of β$_1$AR in the inactive state confirm the *trans* arrangement of M296$^{6.41}$ ($\chi^3 = -169.9 \pm 6.6°$) and show M223$^{5.54}$ in a *gauche* conformation ($\chi^3 = 72.4 \pm 5.6°$) (Supplementary Table 8). The shift towards a *gauche* conformation in the X-ray study of the latter typically relates to the temperature differences between NMR/crystallography but could also be a consequence of the increased thermostabilisation in the X-ray studies. When bound to increasingly activating ligands the ^{13}C shift values decrease both for M223$^{5.54}$ ($\Delta\delta = -0.35$ ppm) and M296$^{6.41}$ ($\Delta\delta = -0.5$ ppm) respectively, indicating increased sampling of *gauche* conformations when bound to full agonist compared to the apo form. (M223 *gauche* population: apo 43%, isoprenaline 57%. M296 *gauche* population: apo 10%, isoprenaline 27%. A simple two state rotamer equilibrium was assumed in these calculations.) The increased sampling of additional conformations is in agreement with the more dynamic behaviour observed for M223$^{5.54}$ and M296$^{6.41}$ in the isoprenaline-bound receptor state as evidenced by the reduced relative signal intensities in Fig. 2. The continuous upfield ^{13}C shift with increasing ligand efficacy as shown in Fig. 1 indicates that adopting an active receptor state relates to increased sampling of *gauche* conformations for M223$^{5.54}$ and deviation from a *trans* rotamer for M296$^{6.41}$. Due to the relatively low energetic rotamer barrier the increased sampling of other conformations that vary from pure *trans* or *gauche* rotamers is also likely and would require the measurement of $^3J_{\mathrm{CC}}$ scalar coupling constants for further assessment.[7]

^{1}H chemical shift data: The ^{1}H chemical shifts of methionine residues are strongly affected by aromatic ring current effects, which displace the methyl signal from its random coil value of 2.13 ppm.[8] In the ligand-bound as well as apo receptor forms the methyl

groups of $M223^{5.54}$ and $M296^{6.41}$ are shifted towards higher field relative to the random coil shift for methionine Hε. This indicates the proximity of aromatic side chains with the aromatic ring normal axis oriented towards the methyl groups, which results in an upfield shift (Supplementary Fig. 14d,e). The dependence on the ligand bound is very small. Accordingly, $M223^{5.54}$ in the apo form is shifted to 1.72 ppm. Inspection of the inactive β_1AR crystal structures confirms the proximity of $M223^{5.54}$ to the side chains of $F299^{6.44}$ (6.5–7.5 Å) and $Y227^{5.58}$ (7–8.5 Å) with the methyl groups facing the ring normal, in agreement with the observed upfield shift (in the structure model of β_1AR bound to cyanopindolol (PDB ID 2VT4) A227 was converted back to Y227 and positioned with the same approximate ring orientation as shown in the β_2AR structure bound to timolol (PDB ID 3D4S)). $M296^{6.41}$ is also in proximity to $Y227^{5.58}$ (4–5 Å) and is shifted upfield to 1.78 ppm, in agreement with the aromatic ring facing the methyl group. ^{1}H chemical shifts for the methyl groups of $M223^{5.54}$ and $M296^{6.41}$ in the inactive form were calculated in MOLMOL[9] based on the Johnson-Bovey equation,[10] for β_2AR bound to the inverse agonists timolol (PDB ID 3D4S) and carazolol (PDB ID 2RH1) (Supplementary Table 9). In all calculations the relative upfield shifts for $M223^{5.54}$ and $M296^{6.41}$ are correctly reproduced (Supplementary Table 9). Differences to the experimental values likely result from sampling additional conformations, which corroborates with the larger deviations observed for $M223^{5.54}$, where our study indicates the sampling of *gauche*/*trans* rotamers. In contrast $M296^{6.41}$ is locked into a *trans* conformation and the calculation for 2RH1 is very close to the experimental data.

Temperature dependence: β_1AR is in conformational exchange between an inactive (I) and active (A) receptor form, I $\rightleftharpoons$ A. For $M223^{5.54}$ and $M296^{6.41}$ this exchange is rapid on the chemical shift timescale. The $M296^{6.41}$ methyl environment in the purely *trans* χ^3 conformation found in the inactive (I) state is in a sterically more constrained environment than in the active state (A). This is in agreement with the cytoplasmic region in the inactive receptor state being less dynamic as revealed by the higher relative intensities compared to the isoprenaline-bound form. Upon reaching the active state the $M296^{6.41}$ environment becomes less constrained. Similar assumptions are made for M82 in β_2AR based on a comparison of active and inactive X-ray structures.[11] Assuming the conformational rearrangement as an entropically driven process lowering the temperature should make the inactive state (I) more favourable and shift the resonance of $M296^{6.41}$ in the direction of the inactive state (I) i.e. downfield. This is indeed observed e.g. when the measurements of isoprenaline-bound β_1AR-MetΔ5-L190M measurements initially carried out at 308 K are repeated at 293 K (Supplementary Fig. 7). The same case applies to $M223^{5.54}$ where upon lowering the temperature the more rigid state with less *trans*/*gauche* interconversion becomes more populated, shifting the $M223^{5.54}$ resonance downfield in the

direction of the inactive state. The observed temperature dependence of $M223^{5.54}$ and $M296^{6.41}$ are further evidence for the existence of the fast exchanging I $\rightleftharpoons$ A equilibrium.

Ternary ligand complexes of β_1AR with nanobody and ligand-free basal activity complex with nanobody

^{13}C chemical shift data: In the ternary active-state complex of β_1AR bound to agonist and nanobody, $M296^{6.41}$ shows a substantial change in ^{13}C chemical shift towards higher field values indicative of adopting an almost pure *gauche* conformation (Supplementary Fig S14a and Supplementary Table 6). Compared to the isoprenaline-bound receptor with $M296^{6.41}$ in the χ^3 *trans* conformation, a shift change of ca. -1.7 ppm is observed upon formation of the ternary isoprenaline complex. Depending on the bound agonist the shifts vary from 16.5 to 16.8 ppm for $M296^{6.41}$, reflecting the changes in the position of the fast exchanging $A^{G-} \rightleftharpoons A^{G+}$ equilibrium. Increasing agonist efficacy seems to further augment the population of the *gauche* rotamer of $M296^{6.41}$. Chemical shift changes for $M223^{5.54}$ indicate a slight increase in the population of *gauche* rotamers in the ternary complexes. However, compared against the ligand-only bound receptor only a relatively small decrease in chemical shift on the order of -0.3 ppm is observed. This is markedly less than for $M296^{6.41}$ and indicates that the *trans* to *gauche* χ^3 rotamer switching of $M296^{6.41}$ is a characteristic feature of β_1AR reaching an active state. Following from the smaller shift change observed for $M223^{5.54}$ between ligand-only and ternary complexes, the variation depending on the agonist bound is also smaller accordingly, with the chemical shift of $M223^{5.54}$ in the ternary complex varying only between 17.0 and 17.05 ppm. Based on the ^{13}C chemical shift values $M223^{5.54}$ in the ternary complexes is still sampling *gauche* and some *trans* conformations that are in rapid exchange with each other. The observations described here for $M223^{5.54}$ and $M296^{6.41}$ apply also to the ligand-free basal-complex of β_1AR bound to nanobody. Adopting a β_1AR active state in the nanobody-bound complexes correlates with a higher population of *gauche* conformations, both for $M223^{5.54}$ and $M296^{6.41}$. As shown in Fig. 5 the increase in nanobody bound β_1AR complex activity correlates with a further increase in the population of the $M296^{6.41}$ χ^3 *gauche* conformation as seen by the ^{13}C upfield shift.

^{1}H chemical shift data: Currently there is no active structure of β_1AR. However, upon activation of class A receptors the conserved residues $Tyr^{5.58}$ (Y227) and $Tyr^{7.53}$ (Y343) adopt highly conserved positions and side chain conformations so that ring current effects on the ^{1}H chemical shifts of $M223^{5.54}$ and $M296^{6.41}$ can be anticipated through investigation of active state β_2AR crystal structures (Supplementary Fig. 14d,e). Following that, in the ternary complexes of the active state of β_1AR, the

aromatic side chain of Y227$^{5.58}$ is expected to change its χ^1 rotameric state, moving closer towards the methyl group of M223$^{5.54}$. The cytoplasmic displacement of TM6 upon activation also moves the aromatic ring system of F299$^{6.44}$ closer towards M223$^{5.54}$. Both orientations have the normal of the aromatic ring system pointing towards the methyl group of M223$^{5.54}$, leading to a strong upfield shift (Supplementary Fig. 14d,e). In addition, Y343$^{7.53}$ on TM7 moves into the gap created by the cytoplasmic displacement of TM6 upon activation and is pointing at TM5. The side-on orientation of Y343$^{7.53}$ facing M223$^{5.54}$ cancels to some extent the upfield shift induced by Y227$^{5.58}$ and F299$^{6.44}$, resulting in an overall moderate upfield shift of M223$^{5.54}$ in agreement with the experimentally observed change from ∼1.72 ppm in the ligand-bound state to ∼1.43 ppm in the ternary complexes (Supplementary Table 9). Similar considerations with regard to F299$^{6.44}$ upon the cytoplasmic displacement of TM6 lead also to a shorter distance to M296$^{6.41}$, where the aromatic ring is in a side-on position. The reduction in distance is likely further accentuated by the change of the conformation of M296$^{6.41}$ from *trans* in the inactive state to *gauche* in the active state, as indicated by the ^{13}C upfield shift of M296$^{6.41}$ (see above). The repositioning of Y227$^{5.58}$ in the active state also results in a shorter distance to M296$^{6.41}$, with the aromatic ring in a side-on arrangement. Both ring current contributions from F299$^{6.44}$ and Y227$^{5.58}$ lead to deshielding, resulting in a downfield shift of M296$^{6.41}$ from 1.72 ppm in the ligand-bound state to 2.02 ppm in the ternary complex. Interestingly the χ^3 conformation of M279$^{6.41}$ in the β_2AR G_s-bound active state structure (PDB ID 3SN6) is *trans*, with the corresponding nanobody-bound active state structure (PDB ID 3P0G) close to *trans*. In contrast, the ^{13}C chemical shift data of the latter complex shows M279$^{6.41}$ at 16.8 ppm, which is in agreement rather with a *gauche* χ^3orientation,[13] and similar to our data on M296$^{6.41}$. ^{1}H chemical shifts representative of the active state of β_1AR were calculated using the structure of β_2AR bound to the full agonist BI-167107 and G_s as a template. However, as suggested by our NMR data the χ^3 rotamer position of Met$^{6.41}$ was adjusted to *gauche* (−67°). The calculations confirm the experimentally observed upfield shift of M223$^{5.54}$ and downfield shift of M296$^{6.41}$ following activation of β_1AR (Supplementary Table 9).

Temperature dependence: In the ternary complex the receptor is in fast exchange between a less and a more active state, $A^{G-} \rightleftharpoons A^{G+}$. It can be assumed that the ternary β_1AR complex with a purely *gauche* conformation of M296$^{6.41}$ as observed in the A^{G+} state is sterically more constrained than when in a state that is sampling *trans* and *gauche* conformations such as in the A^{G-} state. Hence, for an entropically driven conformational rearrangement, lower temperature should see a shift towards an increased population of A^{G+}. As supplementary Fig. 7 shows this is indeed observed with the chemical shifts

of M296$^{6.41}$ showing an upfield shift as the temperature is reduced, indicative of the $A^{G-} \rightleftharpoons A^{G+}$ equilibrium being pushed towards A^{G+}. The same trend is also observed for M223$^{5.54}$, in agreement with a more *gauche* conformation being favoured at lower temperatures. Again, the temperature dependent shift variation backs the existence of the $A^{G-} \rightleftharpoons A^{G+}$ equilibrium.

Supplementary References

1. Tugarinov, V., Hwang, P. M., Ollerenshaw, J. E. & Kay, L. E. Cross-correlated relaxation enhanced ^{1}H-^{13}C NMR spectroscopy of methyl groups in very high molecular weight proteins and protein complexes. *J. Am. Chem. Soc.* 125, 10420–10428 (2003).

2. Kay, L. E. & Gardner, K. H. Solution NMR spectroscopy beyond 25 kDa. *Curr. Opin. Struct. Biol.* 7, 722–731 (1997).

3. Isogai, S., Deupi, X., Opitz, C., Heydenreich, F. M., Tsai, C.-J., Brueckner, F., Schertler, G. F. X., Veprintsev, D. B. & Grzesiek, S. Backbone NMR reveals allosteric signal transduction networks in the β_1-adrenergic receptor. *Nature* 530, 237–241 (2016).

4. Rasmussen, S. G. F., DeVree, B. T., Zou, Y., Kruse, A. C., Chung, K. Y., Kobilka, T. S., Thian, F. S., Chae, P. S., Pardon, E., Calinski, D., Mathiesen, J. M., Shah, S. T. A., Lyons, J. A., Caffrey, M., Gellman, S. H., Steyaert, J., Skiniotis, G., Weis, W. I., Sunahara, R. K., & Kobilka, B. K. Crystal structure of the β_2 adrenergic receptor-Gs protein complex. *Nature* 477, 549–555 (2011).

5. Rasmussen, S. G. F., Choi, H.-J., Fung, J. J., Pardon, E., Casarosa, P., Chae, P. S., DeVree, B. T., Rosenbaum, D. M., Thian, F. S., Kobilka, T. S., Schnapp, A., Konetzki, I., Sunahara, R. K., Gellman, S. H., Pautsch, A., Steyaert, J., Weis, W. I. & Kobilka, B. K. Structure of a nanobody-stabilized active state of the β_2 adrenoceptor. *Nature* 469, 175–180 (2011).

6. London, R. E., Wingad, B. D. & Mueller, G. A. Dependence of amino acid side chain ^{13}C shifts on dihedral angle: Application to conformational analysis. *J. Am. Chem. Soc.* 130, 11097–11105 (2008).

7. Butterfoss, G. L., DeRose, E. F., Gabel, S. A., Perera, L., Krahn, J. M., Mueller, G. A., Zheng, X. & London, R. E. Conformational dependence of ^{13}C shielding and coupling constants for methionine methyl groups. *J. Biomol. NMR* 48, 31–47 (2010).

8. Bundi, A. & Wüthrich, K. ^{1}H-NMR parameters of the common amino acid residues measured in aqueous solutions of the linear tetrapeptides H-Gly-Gly-X-L-Ala-OH. *Biopolymers* 18, 285–297 (1979).

9. Koradi, R., Billeter, M. & Wüthrich K. MOLMOL: A program for display and analysis of macromolecular structures. *J. Mol. Graph* 14:51–55 (1996).

10. Johnson, C. E. & Bovey, F. A. Calculation of Nuclear Magnetic Resonance spectra of aromatic hydrocarbons. *J. Chem. Phys.* 29:1012–1014 (1958).

11. Kofuku, Y., Ueda, T., Okude, J., Shiraishi, Y., Kondo, K., Maeda, M., Tsujishita, H. & Shimada, I. Efficacy of the β_2-adrenergic receptor is determined by conformational equilibrium in the transmembrane region. *Nat. Commun.* 3:1045 (2012).

12. Lebon, G., Warne, T. & Tate, C. G. Agonist-bound structures of G protein-coupled receptors. *Current Opinion in Structural Biology* 22, 482–490.

13. Nygaard, R., Zou, Y., Dror, R. O., Mildorf, T. J., Arlow, D. H., Manglik, A., Pan, A. C., Liu, C. W., Fung, J. J., Bokoch, M. P., Thian, F. S., Kobilka, T. S., Shaw, D. E., Mueller, L., Prosser, R. S. & Kobilka, B. K. The dynamic process of β_2-adrenergic receptor activation. *Cell* 152, 532–542 (2013).

14. Warne, T., Moukhametzianov, R., Baker, J. G., Nehmé , R., Edwards, P. C., Leslie, A. G. W., Schertler, G. F. X., & Tate, C. G. The structural basis for agonist and partial agonist action on a β_1-adrenergic receptor. *Nature* 469, 241–244 (2011).

15. Baker, J. G., Proudman, R. G. W. & Tate, C. G. The pharmacological effects of the thermostabilising (m23) mutations and intra and extracellular (β36) deletions essential for crystallisation of the turkey β-adrenoceptor. *Naunyn. Schmiedebergs. Arch. Pharmacol.* 384, 71–91 (2011).

16. Baker, J. G. A full pharmacological analysis of the three turkey β-adrenoceptors and comparison with the human β-adrenoceptors. *PLoS One* 5(11): e15487 (2010).

17. Sato, T., Baker J., Warne, T., Brown, G. A., Leslie, A. G. W., Congreve, M. & Tate, C. G. Pharmacological analysis and structure determination of 7-methylcyanopindolol-bound β_1-adrenergic receptor. *Mol. Pharmacol.* 88, 1024–1034 (2015).
